# From Chemotherapy to Targeted Therapy: Unraveling Resistance in Acute Myeloid Leukemia Through Genetic and Non-Genetic Insights

**DOI:** 10.3390/ijms26094005

**Published:** 2025-04-24

**Authors:** Shuting Cao, Qiuxia Wang, Ganqian Zhu

**Affiliations:** School of Biomedical Sciences, Hunan University, Changsha 410082, China; sophie00615@gmail.com (S.C.); qiuxiawang147@gmail.com (Q.W.)

**Keywords:** acute myeloid leukemia, drug resistance, chemotherapy, targeted therapy

## Abstract

Acute myeloid leukemia (AML) is a devastating disease characterized by extensive inter-patient and intra-patient heterogeneity. Despite the introduction of intensive chemotherapy in the 1970s as the standard treatment, the development of mechanism-based targeted therapies since 2017 has been broadening the therapeutic landscape. However, both chemotherapy and targeted therapies continue to face the challenges of primary and secondary resistance. This review summarizes the mechanisms underlying resistance to chemotherapy and targeted therapies in AML and discusses the opportunities and challenges brought by the transition from chemotherapy to precision medicine.

## 1. Introduction

Acute myeloid leukemia (AML) is a clonal malignancy of hematopoietic stem/progenitor cells caused by somatic mutations, characterized by uncontrolled proliferation and impaired differentiation of myeloid cells [1,2]. AML can develop at any age but predominantly affects older adults, with the median age of diagnosis of 68 years; over two-thirds of patients are aged 55 and above [3]. Currently, the five-year survival rate for AML patients is approximately 30–35% [4], whereas for those over 60 years old, the cure rate drops to 5–15% [4,5].

Current management of AML relies on intensive chemotherapy (e.g., the “7 + 3” regimen, which consists of 7–10 days of cytarabine and 3 days of daunorubicin), targeted agents, and allogeneic hematopoietic stem cell transplantation (allo-HSCT). However, the traditional “7 + 3” regimen shows limited efficacy, with complete remission (CR) rates of ~50% and a two-year overall survival (OS) of 20% in intermediate-/adverse-risk patients, and its tolerability is limited in older and less fit patients [6,7]. In contrast, CPX-351, a liposomal formulation with a fixed 5:1 cytarabine: daunorubicin molar ratio, optimizes drug delivery by prolonging bone marrow exposure (>24 h) and enhancing leukemic cell uptake [8]. It received FDA approval in 2017 as the first AML-specific liposomal chemotherapeutic agent for frontline therapy in secondary AML based on phase III trial results (NCT01696084). This trial validated its superior efficacy compared to the “7 + 3” regimen in therapy-related AML (t-AML, a high-risk subtype associated with prior cytotoxic therapy) and AML with myelodysplasia-related changes (AML-MRC), demonstrating a 3.61-month median OS improvement (9.56 vs. 5.95 months; one-sided *p* = 0.003) and an 18.8% increase in two-year survival (31.1% vs. 12.3%) [9].

Mechanistic studies have led to the approval of 10 novel targeted therapies by the U.S. Food and Drug Administration (FDA) in the past 8 years (Table 1). These therapies [10] include FMS-like tyrosine kinase 3 (FLT3) inhibitors (e.g., midostaurin, gilteritinib, quizartinib), isocitrate dehydrogenase 1 (IDH1) inhibitors (e.g., ivosidenib, olutasidenib), isocitrate dehydrogenase 2 (IDH2) inhibitor (enasidenib), hedgehog pathway inhibitor (glasdegib), anti-CD33 antibody–drug conjugate (Gemtuzumab Ozogamicin, GO), menin inhibitor (revumenib), and B-cell lymphoma 2 (BCL2) inhibitor (venetoclax). These targeted therapies, either as monotherapies or in combination with chemotherapy, have significantly improved survival outcomes [11].

Beside targeted therapies, the recent approval of oral hypomethylating agents (HMAs)—notably FDA/European Medicines Agency (EMA)-approved oral azacitidine and EMA-approved oral decitabine [12]—has also expanded therapeutic options for AML. These agents have emerged as pivotal components in combination regimens due to their dual advantages: (i) ease of administration, (ii) synergistic activity with other therapies. Clinically, these agents are prioritized for elderly or medically unfit patients ineligible for intensive chemotherapy, typically combined with BCL-2 inhibitors (e.g., venetoclax) or low-dose chemotherapy, with proven efficacy in improving clinical outcomes across multiple trials [13].

Although targeted therapies offer reduced off-target effects compared to traditional chemotherapy, their specificity on a single target renders them vulnerable to the development of resistance [14]. Consequently, relapse remains a major challenge in AML management. This review summarizes the mechanisms driving resistance to chemotherapy and targeted therapies, providing insights into strategies for optimizing treatment approaches.

## 2. Chemotherapy Resistance Mechanisms

**Figure 1:**
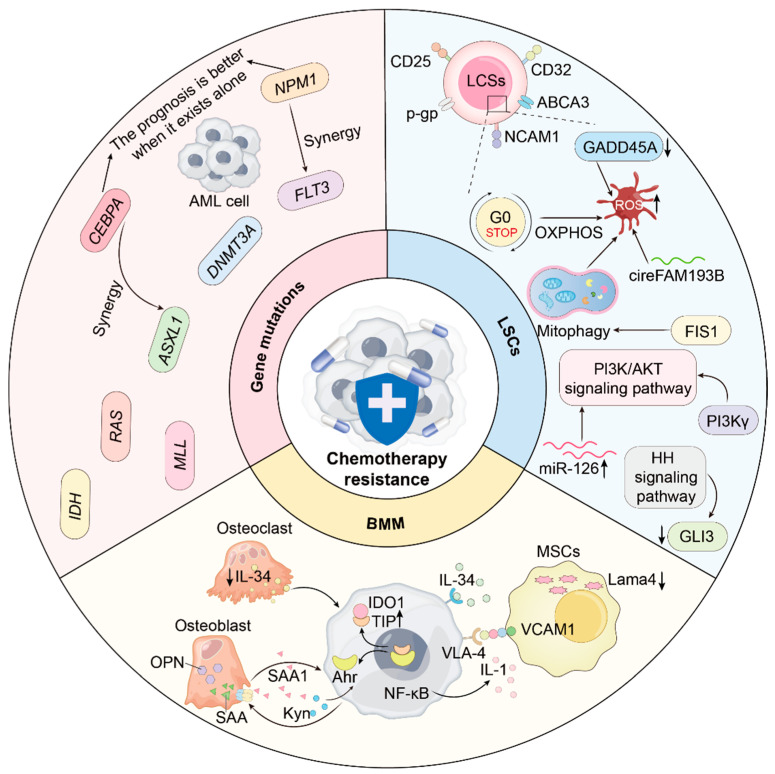
Chemotherapy resistance mechanisms in AML, divided into three main categories: genetic mutations, leukemia stem cells (LSCs), and the bone marrow microenvironment (BMM). Genetic mutations (e.g., *MLL*, *FLT3*-*ITD*, *DNMT3A*) alter cell proliferation, apoptosis, and survival signaling, contributing to resistance. LSCs, with their self-renewal and drug resistance properties, serve as a key source of resistance. The BMM exacerbates resistance through immune evasion, cell signaling, and nutrient support mechanisms. ↑: upregulation; ↓: downregulation.

Chemotherapy can induce complete remission (CR) in most AML patients; however, approximately two-thirds of these patients relapse within 18 months due to drug resistance, which significantly limits treatment efficacy [15]. Chemotherapy resistance is associated with a range of factors, including both genetic and non-genetic mechanisms such as epigenetic changes, cellular states (here we discuss leukemia stem cells (LSCs)), metabolic reprogramming, and alterations in the bone marrow microenvironment [16] (Figure 1).

### 2.1. Gene Mutations and Clonal Heterogeneity

Clonal heterogeneity is a key contributor to chemotherapy resistance and relapse in AML. Pediatric AML, which exhibits lower genetic heterogeneity than adult AML, has a significantly higher five-year survival rate (~68% vs. <30%) [17]. Single-cell sequencing and deep genomic analysis have revealed that AML follows a dynamic clonal evolution model, where early mutations are present in most cancer cells and are referred to as clonal mutations, whereas late mutations are confined to subpopulations of cancer cells, termed subclonal mutations [18]. Relapse occurs when chemotherapy fails to eradicate dominant clones or rare subclones [19,20]. A recent study indicates that resistant clones may preexist before treatment and drive relapse without acquiring additional mutations [21]. Interestingly, chemotherapy stress not only shapes the clonal evolution of cancer cells but also promotes the expansion of hematopoietic stem/progenitor cells (HSPCs) harboring age-related TP53 heterozygous mutations, predisposing patients to t-AML [22].

During AML progression, mutations in epigenetics regulators (e.g., DNA methylation-related genes *DNMT3A*, *TET2*, and *IDH1/2*), RNA splicing factors (e.g., *SRSF2*, *U2AF1*, and *SF3B1*), and chromatin modifiers (e.g., *ASXL1*, *EZH2*, and *BCOR*) often occur early [18], affecting self-renewal and differentiation, whereas mutations in signaling pathway genes (e.g., *NRAS*, *KRAS*, *KIT*, *PTPN11*, and *FLT3*) arise later [18]. As somatic mutations can be readily assessed with current techniques, the latest clinical prognostic standards for AML (ELN 2022) [23] primarily rely on genetic profiling, which remains vital for predicting chemotherapy response. Certain mutations are closely associated with chemotherapy (Table 2). Some of them are also associated with poorer survival in elderly AML patients treated with intensive chemotherapy [24].

Tumor protein (*TP53*) mutations are one of the worst poor prognosis markers of AML, often found coexisting with complex karyotypes, disrupting DNA damage repair and apoptosis, thus rendering AML cells resistant to chemotherapy-induced apoptosis [25]. The enhanced genomic instability induced by the *TP53* mutation accelerates the generation and expansion of resistant clones [22]. Mutant *TP53* also synergizes with oncogenic *NRAS* to induce systemic inflammation by downregulating GATA-binding protein 2 (*GATA2*), which downregulates inflammatory gene expression. Inflammation is involved in chemoresistance and further worsens outcomes [26]. Additionally, elderly patients with AML have an increased probability of RAS pathway activation when compared with their younger counterparts [27]. *RAS* mutant clones were shown to be selected by chemotherapy [10].

Rearrangements of lysine methyltransferase 2A (*KMT2A*, also known as mixed lineage leukemia (*MLL*)) are prevalent in about 23% of t-AML, which is characterized by heterogeneity and chemotherapy resistance [22]. The fusion proteins (e.g., MLL-AF9) typically activate the *HOXA9* and *MEIS1* genes through DOT1L-mediated abnormal H3K79 histone methylation [28,29]. *MLL* rearrangements also enhance cell invasiveness by upregulating EMT-related genes, such as Snail family transcriptional repressor (*SNAIL*) and Twist family bHLH transcription factor 1 (*TWIST*) [30]. These signature genes activated by *MLL* fusion prevent AML cells from chemotherapy-induced cell death and contribute to disease relapse.

FMS-like tyrosine kinase 3 with internal tandem duplication (*FLT3*-ITD) mutation is a key driver of acute leukemia progression [31], strongly associated with poor survival and chemotherapy resistance [32]. Its acquisition at relapse is an independent prognostic factor for chemotherapy failure [31,33]. In most patients with *FLT3*-ITD at diagnosis, the mutation persists at relapse, with an increased allele burden, suggesting clonal expansion of the *FLT3*-ITD subclone that dominates relapse [31].

In addition to *FLT3*-ITD, *IDH1/2* mutations also play a critical role in chemoresistance. *IDH1/2* mutations confer neomorphic activity that enables the production of (R)-2-hydroxyglutarate (R-2-HG), an oncometabolite that accumulates to supraphysiological levels. R-2-HG competitively inhibits α-ketoglutarate (α-KG)-dependent dioxygenases, including TET2 and Jumonji domain-containing histone demethylases, resulting in widespread epigenetic dysregulation and promoting leukemic transformation [34]. Acquired early in leukemogenesis, *IDH1/2* mutations are stably retained throughout disease progression, contributing to chemoresistance by enabling the survival of leukemic stem cells and driving disease relapse [35].

DNA hypomethylation is juxtaposed with focal promoter hypermethylation, alterations strongly associated with increased relapse rates and reduced OS rates [36]. These mutations disrupt normal hematopoietic stem cell (HSC) differentiation, promoting transformation into chemotherapy-resistant LSCs that underlie disease persistence and recurrence [37]. Cells carrying DNA methyltransferase 3 alpha (*DNMT3A*) mutations demonstrate resistance to conventional chemotherapy [38]. The *R882H* mutation, in particular, may decrease daunorubicin sensitivity by activating the nuclear factor erythroid 2-related factor 2 (NRF2)/NAD(P)H quinone dehydrogenase 1 (NQO1) pathway, which regulates cell proliferation and apoptosis [39]. Furthermore, the *R882* mutation synergizes with nucleophosmin 1 mutated cytoplasmic (*NPM1c*) and *FLT3-ITD* mutations to enhance resistance by upregulating anti-apoptotic genes and disrupting chromatin remodeling [40]. Additionally, *DNMT3A* mutations promote Twist family bHLH transcription factor 1 (TWIST1) expression, facilitating extramedullary infiltration of AML cells, thereby compromising treatment efficacy [38]. The clinical impact of *DNMT3A* mutations exhibits age-related heterogeneity, with the *R882* variant conferring particularly adverse outcomes in younger patients [41], while the functional consequences of non-hotspot mutations remain an active area of investigation.

Likewise, additional sex comb-like 1 (*ASXL1*) mutations are associated with chemotherapy resistance and poor prognosis [42]. *ASXL1* truncation mutations disrupt Polycomb Repressive Complex 2 (PRC2)-mediated H3K27 methylation, leading to DNA and histone demethylation, which sustains the undifferentiated and proliferative state of LSCs [43]. Furthermore, *ASXL1* mutations can synergistically accelerate AML progression, especially in the presence of other mutations. For example, in the presence of CCAAT/enhancer-binding protein alpha (*CEBPA*) mutations, the ASXL1-G643W variant accelerates AML progression and chemotherapy resistance by downregulating key leukemia-related pathways, including ribosome biosynthesis, regulation of DNA damage response via p53 mediators, and immune activation [42].

Comparative genomics and epigenomics studies on paired AML samples at diagnosis and relapse revealed that genetic and epigenetic alterations typically progress following independent kinetics only with some intermediate situations [16,44]. Certain mutations (e.g., *DNMT3A R882H*) not only drive the clonal evolution of AML but also enhance chemoresistance by modulating chromatin accessibility [39]. While mutant clonal heterogeneity alone does not necessarily correlate with prognosis, increased epigenetic heterogeneity may portend a poorer outcome [44]. As only a few genetic events are conclusively tied to relapse, the importance of non-genetic mechanisms is drawing growing attention.

### 2.2. Leukemia Stem Cells (LSCs) and Multidimensional Networks

LSCs reside at the apex of the AML’s hierarchy structure and share features with HSCs, including self-renewal and a quiescent cell cycle [45]. These properties make LSCs a major non-genetic source of resistance and relapse [37]. Indeed, LSC-associated signature genes are substantially enriched in post-chemotherapy relapse samples [46]. Many of them are directly involved in chemo-resistance [47].

For instance, CD32 and CD25 regulate LSC quiescence and resistance [48], while neural cell adhesion molecule 1 (NCAM1/CD56) supports LSC survival via MAPK [49]. LSCs exhibit strong bone marrow repopulation potential and drug resistance, enabling them to rapidly expel chemotherapeutic agents via ATP-binding cassette subfamily B member 1 (*ABCB1*)-mediated efflux, thereby diminishing the effectiveness of treatment [50,51]. This drug efflux capacity plays a crucial role in chemotherapy resistance and minimal residual disease in AML [51]. Notably, in pediatric AML, ATP-binding cassette subfamily A member 3 (*ABCA3*) overexpression in LSC also contributes to chemotherapy resistance [52,53]. Furthermore, studies have shown that drug transporters, when expressed on cells with stem cell-like phenotypes, can confer drug resistance, thereby facilitating the selection and expansion of these cells during treatment [54]. Thus, characterization of LSC markers could offer potential targets to overcome chemoresistance [55].

Unlike more mature, glycolysis-dependent AML blasts, quiescent LSCs demand less energy and rely on mitochondrial oxidative phosphorylation (OXPHOS) with low reactive oxygen species (ROS) levels, a property utilized as a sorting marker to isolate LSCs from blasts [56,57,58]. AML cells fueled by OXPHOS often resist chemotherapy [59]. Metabolism pathways are intimately linked to drug resistance. For example, the loss of growth arrest and DNA-damage-inducible 45 alpha (*GADD45A*) increases resistance to ferroptosis by reducing ROS levels [60]. Moreover, circFAM193B interacts with arginine methyltransferase (PRMT6) to enhance H3R2me2a modification, thus activating the transcription of lipid peroxidation factor (ALOX15). It is downregulated in LSCs to maintain the balance of metabolism and redox under chemotherapy and supports survival [61]. In newly diagnosed AML, LSCs primarily utilize amino acid metabolism, but in relapse, they shift to fatty acid metabolism to compensate for OXPHOS and meet elevated energy demands [57]. Mitochondrial fission 1 (FIS1) is also upregulated in LSCs, promoting mitophagy to preserve stemness [58] and associating with poor outcome [62].

Multiple signaling pathways regulate LSC function [63]. PI3K/AKT activation promotes LSC drug resistance by elevating miR-126 [64], and phosphoinositide 3-kinase gamma (PI3Kγ)-AKT signaling promotes nuclear factor erythroid 2-related factor 2 (NRF2) nuclear accumulation, which induces 6-phosphogluconate dehydrogenase (PGD) and the pentose phosphate pathway, thereby maintaining LSC stemness [65]. Additionally, the maintenance of LSCs has been shown to be dependent on Hedgehog (HH) signaling [66]. Hedgehog-glioma-associated oncogene homolog (HH-GLI) pathway dysregulation can modify cell proliferation and survival by lowering GLI3 [67].

Elucidating LSC biology has helped illuminate AML’s non-genetic chemo-resistance mechanisms. Targeting the specialized metabolic, epigenetic, and signaling features of LSCs in combination with chemotherapy may significantly improve therapeutic efficacy.

### 2.3. Bone Marrow Niche and Stromal Components

The bone marrow microenvironment (BMM), consisting of stromal and hematopoietic cell interaction, is crucial for maintaining the survival and function of hematopoietic cells. This specialized milieu is known as the *niche*. HSCs primarily self-renew within the bone marrow (BM) niche, while other blood cells use the spleen or lymph nodes as their niche. Similar to the preference of quiescent HSCs for the endosteal BM niche, LSCs at the leukemia initiating stage rely on wingless-type MMTV integration site family (WNT) signaling to engraft in the endosteal region of BM and then tend to colonize the central BM niche as the disease worsens [68]. HSCs and LSCs located in the endosteal niche often show more chemoresistance [69]. The BMM grants a ‘safe haven’ for LSCs, protecting them from chemotherapy via providing survival and proliferative signals, and enabling the following selection for secondary genetic mutations [70,71]. AML cells, in turn, manipulate the BM niche to favor their own survival. For instance, AML blasts release BMP6, which induces ID1 expression in BMM cells, especially mesenchymal stem cells (MSCs). ID1 then interacts with ring finger protein 4 (RNF4), an E3 ubiquitin ligase, curtails specificity protein 1 (SP1) ubiquitination, and increases the expression and secretion of angiopoietin-like protein 7 (ANGPTL7), thus advancing AML [72]. Here, we discuss the roles of specific BMM stromal cell populations such as MSCs, osteoblasts, and osteoclasts in chemoresistance.

#### 2.3.1. Mesenchymal Stem Cells (MSCs)

Abnormal proliferation of myeloid cells, malignant or otherwise, can cause the overproduction of proinflammatory cytokines like IL-6 and TNFα [73,74]. In myelodysplastic syndromes (MDSs), upregulated pro-inflammatory cytokines appear in bone marrow MSCs [75]. MDS cells can reprogram MSCs in the niche, activating cytokine production that propagates disease [76]. Co-culture experiments indicate that MSCs respond to AML cells by increasing the expression of IL-6, CCL2, and vascular cell adhesion molecule-1 (VCAM-1). In an autocrine manner, VCAM-1 can activate NF-κB via its receptor, very late antigen-4 (VLA-4, α4β1 integrin), to promote AML cells survival and confer chemoresistance on them [77]. High levels of VLA-4 on AML cells can also lessen chemotherapy sensitivity by binding fibronectin in the bone marrow [78,79]. MSC-derived VCAM-1’s role in AML still needs deeper investigation. Moreover, AML cells form connexin-43 (CX43)-based gap junctions with MSCs, and this physical connection can reprogram MSC into AML-MSC via altering its transcriptomes and secretomes in a pro-leukemia direction. Interrupting AML-MSCs crosstalk with lercanidipine can heighten the efficacy of both chemotherapy and targeted agents [80].

Interestingly, not all AML-MSC-mediated effects are pro-tumor. Knockout of Lama4, a key cell matrix adhesion molecule upregulated in mice AML-MSCs [81], impairs hematopoiesis recovery accompanied by alterations in the BM niche after irradiation. Unexpectedly, inhibiting or silencing Lama4 in human and mouse MSCs promotes AML progression and chemoresistance, underscoring the complex effects of MSC remodeling in AML [82].

#### 2.3.2. Osteoblasts and Osteoclasts

As critical components of the HSC niche, both osteoblasts and osteoclasts play essential roles in maintaining normal hematopoiesis [83]. Notably, expression of a constitutively active β-catenin mutant in osteoblasts has been shown to shift HSC differentiation toward the myeloid lineage and promote AML development. Meanwhile, transplantation of wild-type bone marrow into lethally irradiated mice expressing the β-catenin mutant was observed to induce AML [84]. These findings indicate osteoclasts as a niche component for both LSCs and HSCs and suggest their potential involvement in chemoresistance. The osteoblast-secreted factor osteopontin (OPN), which normally maintains HSC quiescence, is frequently overexpressed in AML bone marrow and correlates with poor clinical outcomes [85]. While its putative role in chemoresistance warrants further investigation, emerging evidence suggests complex bidirectional interactions between AML cells and factors secreted by osteoblasts. AML cells also exploit peripheral serotonin signaling pathways to alter osteoblast function, forming a self-reinforcing cycle that sustains leukemic proliferation [86]. Mechanistically, AML-derived kynurenine (Kyn) acts as an oncometabolite and 5-hydroxytryptamine receptor 1B (HTR1B), which is a Gi-coupled receptor ligand on osteoblasts, triggering the secretion of serum amyloid A (SAA). SAA then activates indoleamine 2,3-dioxygenase 1 (IDO1), the rate-limiting enzyme for kynurenine synthesis, redirecting tryptophan metabolism toward the kynurenine pathway. This positive feedback loop amplifies Kyn production, thereby promoting AML progression [86]. Furthermore, SAA1-mediated IDO1 upregulation has been demonstrated to accelerate AML progression through immune evasion and tolerance [87]. Notably, clinically relevant studies reveal a characteristic reduction in osteoblast numbers within patient bone marrow and preservation of osteoblast populations appear to impede leukemia progression [88]. These paradoxical findings highlight the need to elucidate the precise mechanisms through which AML cells interact with and reprogram osteoclasts to establish a pro-leukemic and chemo-resistant microenvironment.

Preclinical AML models have revealed that Tsc1 deficiency in osteoclasts triggers substantial interleukin-34 (IL-34) secretion. IL-34 then inhibits the Ras-ERK1/2 signaling pathway via binding to the myeloid receptor 2 (TREM2) on AML cells, promoting their differentiation and thus suppressing AML progression [89]. Conversely, ablation of IL-34 in mouse models accelerates AML pathogenesis [89]. While preclinical evidence suggests therapeutic potential for IL-34-mediated regulation, its clinical efficacy and translational applicability necessitate rigorous validation in human trials.

### 2.4. Optimized Chemotherapy Strategies

The clinical utility of cytarabine and daunorubicin as backbone agents in AML therapy is frequently undermined by complex resistance mechanisms. This therapeutic challenge has driven the development of fludarabine-based combination strategies, where the fluorinated purine analog fludarabine enhances cytarabine efficacy through dual mechanisms: intracellular Ara-CTP accumulation and DNA repair inhibition [90]. The FLAG regimen (fludarabine, cytarabine, G-CSF) has demonstrated particular efficacy in relapsed/refractory AML, showing favorable response rates and tolerability profiles, especially in BM transplantation candidates [91]. Further refinement through idarubicin incorporation (FLAG-Ida) has established this as a standard induction approach for younger high-risk AML and MDS patients, achieving superior CR rates with reduced relapse incidence [92].

Molecular subtype-specific therapeutic strategies show particular promise in core-binding factor AML (CBF-AML), which is characterized by frequent co-occurring mutations in *FLT3*, *c-KIT*, *RAS*, and other genes that confer adverse prognosis [10]. Importantly, the FLAG-GO regimen (fludarabine/cytarabine/gemtuzumab ozogamicin) not only leverages CD33-targeted synergy to improve clinical outcomes [93], but also appears to mitigate the poor prognostic impact of high-risk molecular markers, including *c-KIT* mutations (VAF ≥ 25%), *TET2* mutations, and *FLT3*-ITD, that typically predict inferior survival under conventional “7 + 3” chemotherapy [10]. This suggests FLAG-GO’s enhanced antileukemic activity may overcome the detrimental effects of these molecular aberrations. The unique mechanistic profile of cladribine, inducing mitochondrial-mediated cell death in both proliferating and quiescent populations, further supports its inclusion in regimens like cladribine, cytarabine, and granulocyte colony-stimulating factor (CLAG) [94]. Ongoing investigation into the chemoresistance-overcoming properties of these optimized combinations will be crucial for advancing precision therapy paradigms in AML.

## 3. Targeted Therapy and Resistance Mechanisms

For decades, frontline AML treatment followed a one-size-fits-all strategy of cytarabine combined with anthracycline (“7 + 3” regimen) [95]. Since 2012, hypomethylating agents (HMAs) like azacitidine and decitabine have emerged as alternatives for patients unfit for intensive chemotherapy [96]. With the growing understanding of AML biology, precision medicine is ushering in a new era of AML treatment, shifting from chemotherapy toward targeted therapies tailored to the genetic features of an individual patient. Over the past 8 years, the FDA has approved 10 inhibitors targeting different drivers (e.g., BCL2, FLT3, IDH1/IDH2, and Menin) of AML. These agents are currently integrated into clinical practice across multiple settings, as adjuncts of chemotherapy in first-line treatment, salvage therapy for refractory or relapsed disease, and maintenance therapy during remission. Their introduction has necessitated revisions to traditional clinical risk stratification systems. A notable example is the reclassification of *FLT3*-ITD mutations by the European LeukemiaNet (ELN) guidelines. The *FLT3*-targeting multi-tyrosine kinase inhibitor (TKI) midostaurin has demonstrated benefit in *FLT3*-ITD patients regardless of variant allelic frequency (VAF), prompting the ELN to categorize all *FLT3*-ITD mutations as of intermediate risk irrespective of VAF or co-existing *NPM1* mutations in 2022 [23].

This evolving landscape suggests that further updates to risk stratification are likely imminent. While targeted drugs have significantly improved survival and response rates, they share a fundamental limitation with conventional chemotherapy: the imposing of selective pressure on malignant clones, driving inevitable evolution of resistance through resistant clone expansion, epigenetic plasticity, metabolic reprogramming, and microenvironment-mediated protection. Notably, unique on/off-target mutations create an additional dimension of resistance in targeted therapy.

### 3.1. Mutation-Specific Inhibitors

#### 3.1.1. FMS-like Tyrosine Kinase 3 (FLT3) Inhibitors

FLT3, a type 3 receptor tyrosine kinase (RTK) belonging to the FMS-like tyrosine kinase family, is widely expressed on AML blasts. *FLT3*-activating mutations (typically ITD in 25% of AML cases and tyrosine kinase domain (TKD) point mutations in ~5%) result in constitutively activated *FLT3* signaling independent of ligand binding, representing one of the most prevalent driver mutations in AML with an overall incidence of ~30%. Among these mutations, *FLT3*-ITD in the juxtamembrane domain disrupts autoinhibitory steric hindrance [97], while *FLT3*-TKD stabilizes the active kinase conformation [98].

First-generation FLT3 inhibitors (e.g., midostaurin, sorafenib, lestaurtinib), as broad-spectrum tyrosine kinase inhibitors (TKIs), exhibit limited and transient antileukemic activity when administered as monotherapy. However, early reports [99] and the RATIFY trial [100] demonstrated synergy between midostaurin and standard induction chemotherapy, leading to its FDA approval in 2017 as a first-line therapy for *FLT3*-ITD/TKD AML. Despite this advancement, resistance and relapse remain major challenges. Early in vitro studies using the *FLT3*-ITD-expressing BA/F3 cell line treated with mutagen revealed that, unlike other FLT3i (e.g., Sorafenib, SU5614), midostaurin resistance mutations specifically localize to the tyrosine kinase domain 1 (TK1) at position N676 [101]. Clinically, the *N676* missense mutation was identified in relapsed AML patients post midostaurin treatment and this mutation is sufficient to confer resistance to midostaurin in vitro [102]. Beyond this secondary on-target mutation, AML cells can evade FLT3 inhibition through compensatory activation of alternative pathways (e.g., JAK/STAT, PI3K/AKT, MAPK), particularly in cases with co-occurring *JAK* mutations [103]. Analysis of paired samples from the RATIFY trial cohort (*n* = 54 relapsed/refractory *FLT3*-ITD patients) revealed divergent clonal evolution pattens: 54% retained *FLT3*-ITD mutations at progression, whereas 46% became *FLT3*-ITD-negative, suggesting selection of *FLT3*-independent clones under midostaurin pressure. Notably, among mutation-persistent cases, only 11% developed novel on-target *FLT3* mutations, with the N676 variant detected in merely two patients at low VAF (<5%) [104]. These findings collectively indicate that off-target mechanisms, rather than secondary *FLT3* mutations, drive midostaurin resistance in most cases.

Second-generation FLT3i (e.g., gilteritinib, quizartinib, crenolanib) demonstrate enhanced selectivity and potency against FLT3, achieving clinically meaningful monotherapy responses [105]. In the ADMIRAL trial [106], gilteritinib improved CR rates and OS, earning FDA approval in 2018 for relapsed/refractory *FLT3*-mutant AML as monotherapy. Resistance to gilteritinib manifests through temporally distinct mechanisms: early resistance involves bone marrow stromal cell-derived factors (FGF2, FLT3 ligand) that induce aurora kinase B (AURKB)-dependent cell cycle arrest and lipid metabolic reprogramming in surviving leukemic cells [107], while late resistance correlates with the expansion of pre-existing NRAS/KRAS mutant subclones and persistent metabolic adaptations [108]. Notably, *NRAS* mutations alone are insufficient to confer immediate resistance, highlighting the essential role of non-genetic mechanisms such as epigenetic remodeling and the microenvironment in FLT3 inhibitor resistance [107].

Emerging evidence reveals that FLT3 signaling activates transcription factor C/EBPα to promote fatty acid (FA) biosynthesis and desaturation, thereby maintaining lipid homeostasis and conferring adaptation to redox stress in *FLT3*-mutant AML. This lipid redox stress vulnerability renders *FLT3*-mutant cells’ susceptibility to ferroptosis inducers upon FLT3 inhibition. The following studies demonstrate synergistic effects between FLT3 inhibitors and ferroptosis inducers both in vitro and in vivo, suggesting a novel therapeutic strategy to enhance sensitivity or overcome resistance to gilteritinib in *FLT3*-mutant AML [109].

Quizartinib, a type II FLT3i selective for *FLT3*-ITD, received FDA approval in July 2023 as a frontline treatment for *FLT3*-ITD AML in combination with “7 + 3” induction, following a consolidation regimen in combination with cytarabine, and as maintenance monotherapy after consolidation (excluding maintenance post allogeneic orthotopic transplantation (allo-OT)). Distinct from gilteritinib and midostaurin, quizartinib resistance typically arises through acquiring on-target *FLT3* mutations. Relapsed patients receiving quizartinib frequently gain mutations at the activation loop residue D835 or the ‘gatekeeper’ residue F691 on FLT3 (this could occur either on *FLT3*-ITD or FLT3 naive alleles). Both residues are localized on the tyrosine kinase domain of FLT3, and their missense mutations substantially reduce the binding affinity of quizartinib to FLT3 or confer compensatory FLT3 signaling [110,111]. In other words, the acquisition of *FLT3*-TKD mutations confers resistance to quizartinib in *FLT3*-ITD AML cells, as this agent exclusively targets *FLT3*-ITD. Notably, it is possible quizartinib may select for preexisting TKD mutant clones in native FLT3 alleles, but TKD mutant cells do not appear de novo to dominate at relapse.

Beyond target-specific mechanisms, the enhancer of zeste homolog 2 (EZH2), the catalytic subunit of PRC2, shows loss-mediated resistance to both quizartinib and cytotoxic agents, which is not neglectable [112]. Moreover, the bone marrow microenvironment actively promotes resistance through cytokine-mediated signaling (e.g., FGF2, IL-3, GM-CSF), which activates survival pathways that counteract FLT3 inhibition [113].

#### 3.1.2. Isocitrate Dehydrogenase (IDH) Inhibitors

*IDH1* and *IDH2*, which catalyze the conversion of isocitrate to α-ketoglutarate (α-KG) in the tricarboxylic acid (TCA) cycle, are mutated in AML with reported incidence of ~20% [114]. *IDH1* mutations predominantly occur at the *R132* residue, whereas *IDH2* mutations localize to R140 or R172 [115]. These gain-of-function mutations confer neomorphic activity to IDH enzymes, leading to the reduction of α-KG to 2-hydroxyglutarate (2-HG). The resultant pathogenic accumulation of this oncometabolite competitively inhibits α-KG–dependent ten-eleven translocation (TET) dioxygenases, disrupting epigenetic regulation and ultimately impairing granulocytic differentiation [35].

IDH inhibitors (IDHi) selectively target mutant IDH enzymes, suppressing 2-HG production to restore metabolic homeostasis and normalize epigenetic patterns, thereby inducing AML cell differentiation while suppressing proliferation [116,117]. However, therapeutic resistance develops through multiple mechanisms. Isoform switching, manifested as *IDH2* mutation emergence in *IDH1*-mutant patients (or vice versa) at relapse, constitutes a mechanism of secondary resistance [118]. Additionally, acquired mutations in the wild-type IDH allele that does not confer the neomorphic activity disrupt IDHi binding at the IDH–dimer interface, representing another mechanism’s acquired resistance [119].

Although IDHi can induce durable remission [120], primary resistance manifesting as poor clinical response occurs in ~50% of cases [121]. Notably, co-occurring mutations in *RUNX1*, *CEBPA*, *GATA2*, and *NRAS* show a significant association with reduced CR rate [121,122]. Furthermore, primary resistance correlates with an increased mutation burden and upregulated LSC signature genes [119,122]. Intriguingly, LSC signature genes are not enriched in relapsed patients, indicating distinct mechanisms between primary resistance and acquired resistance [121]. Importantly, clone selection of primary resistance-associated mutations in *RUNX1* and *RAS-RTK* pathway genes also contribute to acquired resistance and relapse [121].

AML cells with *IDH* mutations exhibit augmented mitochondrial oxidative metabolism, primarily driven by CEBPα-mediated upregulation of fatty acid oxidation (FAO) [123]. Although IDHi treatment reduces 2-HG accumulation and CEBPα methylation, it fails to completely normalize FAO and OXPHOS levels, thereby promoting metabolic adaptation and resistance [123]. These findings suggest that combining metabolic modulators such as OxPHOS inhibitors with IDHi may represent a promising strategy to enhance therapeutic efficacy.

#### 3.1.3. Menin Inhibitor

The *MLL* gene rearrangement, a recurrent driver event in AML, occurs on chromosome 11q23 and encodes a histone methyltransferase essential for transcriptional regulation and chromatin remodeling [124]. Present in 5–10% of AML cases [125], these rearrangements generate fusion proteins (e.g., MLL-AFF1, MLL-AF9) that induce leukemogenesis through dysregulated transcriptional programs. Revumenib, a potent Menin inhibitor (Menin-i), selectively targets *MLL*-rearranged (*MLL-r*) and *NPM1*-mutated AML. Unlike conventional targeted therapies that directly inhibit mutant oncoproteins (e.g., FLT3 or IDH1/2 inhibitors), revumenib exerts its therapeutic effects by modulating epigenetic regulatory mechanisms in leukemic cells rather than suppressing mutant proteins [126].

Resistance to Menin inhibition is frequently driven by acquired somatic *MEN1* mutations in *MLL*-r or *NPM1c*-mutated AML. These mutations induce recurrent amino acid substitutions (e.g., M327I, G331R, T349M, S160C) that confer cross-resistance to structurally diverse Menin inhibitors, including revumenib [127]. Notably, while revumenib induces myeloid differentiation in *MLL*-r AML—contrasting with its pro-apoptotic effects in acute lymphoblastic leukemia (ALL)—intrinsic resistance persists in certain AML cell lines [128].

Emerging evidence suggests that resistance extends beyond single-gene alterations to encompass dynamic chromatin complex adaptations. Menin inhibition triggers dissociation of the MLL1–Menin complex, enabling compensatory recruitment of the MLL3/4- ubiquitously transcribed tetratricopeptide repeat on chromosome X (UTX) complex. This transcriptional rewiring activates tumor suppressor genes through UTX’s non-catalytic functions (e.g., co-factor recruitment, chromatin structural modulation) rather than its demethylase activity. Consequently, functional impairments in UTX, MLL3, or MLL4 may subvert therapeutic efficacy, representing a novel resistance axis [129].

The clinical application of revumenib requires careful consideration of fusion partner-specific transcriptional networks in relapsed/refractory *MLL*-r AML [130]. For instance, *NUP98*-rearranged AML subtypes (e.g., *NUP98: KMT2A*) lacking Menin-binding domains exhibit inherent resistance. Co-occurring mutations (*ASXL1*, *IDH1*, *BCORL1*) further modulate sensitivity, while *MEIS1*/*HOXA9* overexpression serves as a predictive biomarker for response. In contrast, *RUNX1:ETO* fusions bypass Menin dependency and remain refractory to inhibition [131,132]. Additionally, *MLL* fusion-driven transcriptional reprogramming toward a granulocyte-monocyte progenitor (GMP)-like state contributes to secondary resistance. Remarkably, even upon *MLL* fusion elimination in lineage-converted AML, persistent eleven-nineteen leukemia (ENL/MLLT1)-mediated activation of GMP-associated genes sustains leukemic survival [133]. Further refinement of predictive biomarkers and mechanistic insights into lineage-specific responses will be critical to optimize its clinical utility.

### 3.2. Hedgehog (HH) Signaling Pathway Inhibitor—Glasdegib

The pathogenesis of AML involves dysregulated activation of oncogenic signaling pathways, with recent studies highlighting the critical role of HH signaling in LSC self-renewal. Central to this pathway is Smoothened (SMO), a transmembrane protein that transduces HH signals. Pharmacologic SMO inhibition potently suppresses the leukemia-initiating capacity of AML cells and synergizes with conventional chemotherapy [134].

Resistance to glasdegib in AML is primarily driven by functional loss of the GLI3 repressor (GLI3R), a tumor-suppressive isoform that epigenetically suppresses HH pathway activity. In over 80% of AML cases, promoter hypermethylation silences GLI3 expression, resulting in ligand- and SMO-independent activation of downstream signaling [135,136]. This epigenetic alteration explains the limited clinical efficacy of SMO inhibitors (SMOi) in the majority of AML patients. Hypomethylating agents, such as decitabine, restore GLI3 expression, re-establishing pathway regulation and resensitizing SMOi-resistant leukemic cells [137]. These findings position GLI3R as a potential biomarker for predicting SMOi responsiveness and for pharmacodynamic monitoring during SMOi-based therapies.

Zebrafish models have revealed an alternative resistance mechanism mediated by histone deacetylase 6 (HDAC6). Both HH pathway hyperactivation and HDAC6 overexpression drive pathological expansion of HSPCs, a phenotype that is selectively reversed by HDAC6 inhibition rather than HH pathway blockade [138]. These findings position HDAC6 as a tractable target to augment the efficacy of SMO-directed therapies.

### 3.3. Apoptosis-Targeted Therapy—Venetoclax

Escape from apoptosis is a hallmark of tumorigenesis and a major driver of drug resistance. In AML, this process is frequently mediated by the upregulation of anti-apoptotic Bcl-2 family proteins and dysfunction of the p53 pathway [139,140]. BCL2, a key anti-apoptotic protein, inhibits the activation of pro-apoptotic BAX and BAK, thereby preserving mitochondrial membrane integrity and blocking apoptosis [141]. In AML, BCL2 overexpression or mutations often co-occur with genetic alterations such as *FLT3*-ITD and *NPM1* mutations, highlighting its synergistic role in leukemogenesis [142]. Venetoclax (ABT-199), a highly selective BCL2 inhibitor, directly binds to BCL2, displacing pro-apoptotic proteins and inducing apoptosis in malignant cells [139].

Despite its clinical efficacy, venetoclax-based therapies are not curative, and resistance remains a significant challenge. A key resistance mechanism involves the upregulation of alternative anti-apoptotic proteins, particularly MCL-1, which sequesters pro-apoptotic factors like BIM and counteracts venetoclax-induced apoptosis [143]. MCL-1’s unique metabolic and signaling properties further enhance its resistance to apoptosis [144,145]. Additionally, p53 dysfunction, commonly observed in refractory AML, reduces mitochondrial priming and impairs the apoptotic response to BCL2 inhibition [139,146]. Targeting p53 activation in combination with venetoclax may therefore improve therapeutic outcomes [144].

Metabolic reprogramming also contributes to venetoclax resistance. Leukemia cells can upregulate nicotinamide metabolism, altering the NAD+/NADH ratio to sustain mitochondrial function and evade apoptosis [147]. Furthermore, LSCs exhibit increased reliance on OXPHOS and adaptive mitochondrial dynamics, enabling them to survive venetoclax treatment [148,149].

Dysregulated RNA splicing has recently been identified as a novel mechanism underlying venetoclax resistance. Aberrant splicing of apoptotic and survival genes generates splice variants that bypass apoptotic pathways, while upregulation of *MAPK*-related genes exacerbates the resistance phenotype [150,151]. These findings underscore the complexity of venetoclax resistance and highlight the need for multi-targeted therapeutic strategies.

### 3.4. Epigenetic Inhibitors—HMAs (Azacitidine and Decitabine)

DNA methyltransferases (DNMTs) catalyze the addition of methyl groups to cytosine residues, playing a critical role in the epigenetic regulation of gene expression [152]. In AML, overexpression or mutations of *DNMT1*, *DNMT3A*, and *DNMT3B* frequently result in aberrant methylation patterns, leading to dysregulation of key genes that drive leukemogenesis [153]. Hypomethylating agents (HMAs), such as azacitidine (AzaC) and decitabine (DAC), reverse pathological methylation, restore normal gene expression, and induce differentiation or apoptosis in leukemic cells [154]. However, the long-term efficacy of HMAs is often limited by the development of resistance.

Resistance to HMAs is driven by multiple mechanisms, especially in cellular mutations and metabolic adaptations. For instance, AzaC treatment can induce P-glycoprotein (P-gp) expression and efflux activity in AML cells. Although AzaC is not a direct P-gp substrate, this adaptation confers cross-resistance to other P-gp substrates and glutathione S-transferase substrates [155]. Additionally, reduced activity or expression of metabolic enzymes, such as uridine-cytidine kinase (UCK1/2) [156] and deoxycytidine kinase (DCK) [157], impairs the activation of AzaC and DAC, while cytidine deaminase (CDA) inactivates DAC by degrading its metabolites, shortening its half-life [158,159]. Upregulation of SAMHD1 further contributes to resistance by regulating intracellular levels of DAC-triphosphate (DAC-TP) [160]. Beyond intrinsic cellular mechanisms, the quality and quantity of bone marrow-infiltrating T cells have also been implicated in modulating treatment outcomes and patient resistance [161].

### 3.5. Immunotherapy

**Figure 2:**
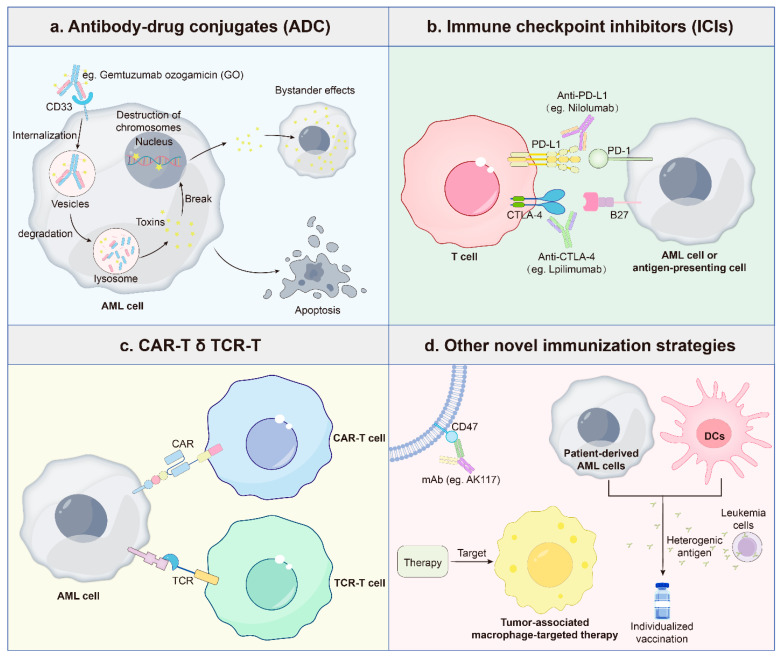
Types and mechanisms of immunotherapy in AML. (**a**) Antibody–drug conjugates (ADCs): CD33-targeted ADCs (e.g., GO) represent the clinically validated immunotherapeutic backbone in AML, utilizing monoclonal antibodies to deliver cytotoxic payloads (e.g., calicheamicin) selectively to leukemic blasts. Next-generation ADCs targeting CD123, FLT3, and CLEC12A are in clinical development, aiming to overcome antigenic heterogeneity and myeloid toxicity limitations. (**b**) Immune checkpoint inhibition (ICI): Checkpoint blockade therapies targeting PD-1/PD-L1 or CTLA-4 pathways aim to restore antitumor immunity by disrupting inhibitory interactions between leukemic cells and immune effector cells. However, their efficacy is highly dependent on tumor mutational burden and baseline T-cell infiltrates [175]. (**c**) Engineered cellular therapies: Chimeric antigen receptor T-cell therapy (CAR-T) platforms, targeting surface antigens and TCR-T therapies recognizing intracellular neoantigens, leverage genetically modified T-cells for AML-specific cytotoxicity. Despite challenges from antigen escape and on-target/off-leukemia effects, early clinical data show durable remissions in high-risk subsets [176]. (**d**) Emerging immunomodulatory strategies: Monoclonal antibodies (mAbs), cancer vaccines, and immune modulators represent novel approaches to amplify anti-AML immunity through distinct mechanisms. Most remain in preclinical or phase I/II testing with combinatorial potential.

Immunotherapy has emerged as a pivotal treatment strategy for AML and other hematologic malignancies (Figure 2). Among antibody-based approaches, CD33-targeted therapy holds particular significance due to its clinical validation. CD33, a glycoprotein highly expressed on myeloid progenitor cells, is detectable in approximately 90% of AML cases [162]. The CD33-directed antibody–drug conjugate gemtuzumab ozogamicin (GO)—a humanized monoclonal antibody conjugated to the cytotoxic calicheamicin—exemplifies this strategy, selectively inducing apoptosis in CD33-expressing leukemic cells [163].

As the first FDA-approved immunotherapeutic agent in AML, GO has established a critical proof-of-concept for antigen-targeted therapies. Nevertheless, its clinical efficacy is constrained by intrinsic and acquired resistance mechanisms. Overexpression of P-glycoprotein (P-gp) drives resistance in AML cell lines and primary cells, whereas multidrug resistance protein 1 (MRP1) plays a subsidiary role [164,165]. Concurrently, upregulation of anti-apoptotic proteins such as BCL-2 and BCL-xL attenuates GO-induced cytotoxicity, further contributing to therapeutic failure [166].

Genetic variations in CD33 also influence therapeutic outcomes. The germline CD33 single nucleotide polymorphism (SNP) rs12459419 is strongly associated with GO response. Patients with the CC genotype exhibit a significantly lower relapse rate (26% vs. 49%, *p* ≤ 0.001) due to enhanced CD33-GO binding, whereas CT or TT genotypes are linked to poorer responses and relative resistance [167]. These findings highlight the importance of genotype-based personalized therapy in AML.

Beyond antibody-based approaches, immune checkpoint inhibitors (e.g., Programmed cell death protein 1 (PD-1)/Programmed cell death ligand 1 (PD-L1), and cytotoxic T-lymphocyte-associated protein 4 (CTLA-4) inhibitors) have shown limited efficacy in relapsed/refractory AML, primarily due to immune evasion mechanisms such as PD-L1 upregulation and Treg-mediated immunosuppression [168,169]. Similarly, T-cell-based immunotherapies face challenges including antigen loss, T-cell dysfunction, and tumor microenvironment (TME) remodeling [170]. Personalized vaccines, though still in early clinical trials, offer potential by inducing leukemia-specific T-cell expansion and reducing relapse risk [171]. However, antigenic heterogeneity and immune-editing mechanisms may compromise vaccine efficacy [172], mirroring resistance patterns observed in other immunotherapies.

Recent advances focus on modulating the immune microenvironment to enhance therapeutic responses. Anti-CD47 monoclonal antibodies and tumor-associated macrophage (TAM)-targeting strategies have shown promise in preclinical and clinical studies [173,174]. When combined with immunotherapy, targeted therapy, and novel drug delivery systems, these approaches may significantly improve treatment outcomes and patient quality of life.

## 4. Integrative Perspectives and Future Directions

Among AML subtypes, acute promyelocytic leukemia (APL) historically represented one of the most lethal forms. Prior to the advent of modern induction therapies, the CR rate in APL remained critically low at approximately 13% [177]. The therapeutic landscape was revolutionized by the clinical implementation of all-trans retinoic acid (ATRA) and arsenic trioxide (ATO), which synergistically improved 5-year OS to over 90% in contemporary cohorts [178]. This paradigm shift has established APL as the AML subtype with the most favorable prognosis, providing critical insights for developing therapeutic strategies in other AML variants. Given the inherent complexity of clinical heterogeneity and clonal evolutionary dynamics, elucidating drug resistance mechanisms emerges as a cornerstone for advancing novel therapeutic agents, repurposing existing modalities, and optimizing combinatorial approaches.

Multi-omics analytical frameworks are redefining our capacity to decipher and circumvent AML resistance mechanisms. Single-cell multi-omics profiling has recently unveiled clonal dynamics in relapsed/refractory AML, identifying actionable targets such as polo-like kinase 1 (PLK1) inhibition with volasertib for proliferative subclones and CD36-neutralizing antibodies for metabolic adaptation [179]. Furthermore, phosphoproteomic signatures coupled with kinase activity mapping have enabled prediction of chemoresistance patterns, nominating AURKB kinase activation as a putative predictive biomarker [180]. In addition, the convergence of spatial transcriptomic mapping [181] and genome-scale CRISPR screening [182] provides a powerful paradigm to deconvolute multidrug resistance in AML, offering high-resolution insights into clonally heterogeneous drug evasion strategies and therapeutically targetable compensatory signaling networks. These findings bridge the gap between molecular discovery and clinical application, as evidenced by the ongoing Beat AML Master Trial (NCT03013998), employing real-time drug sensitivity testing to guide therapy [183].

The elucidation of prior resistance mechanisms has guided the development of novel molecular targeted therapies, exemplified by the fourth-generation FLT3 inhibitor FF-10101 that specifically overcomes FLT3 mutations (including quizartinib-resistant mutations) [184]. Insights from vorasidenib’s success in targeting IDH1/2 mutations in gliomas [185] suggest that similar dual-target inhibition strategies could address metabolic adaptation-mediated resistance in AML. Building upon these molecular approaches, combination therapies demonstrate enhanced efficacy through FLT3-MEK co-inhibition to suppress compensatory bypass signaling and IDH inhibitor–venetoclax pairing to synergistically induce metabolic stress [186]. Independently, bispecific antibodies (e.g., FLT3-CD3 [187]) and CAR-T cell therapy [176] also emerge as alternative mechanisms to overcome resistance in relapsed and refractory patients. These therapeutic advances are further substantiated through clinical implementation strategies encompassing circulating tumor DNA (ctDNA)-guided sequential treatment optimization, augmented by approaches employing epigenetic modulation and microenvironmental remodeling (e.g., the C-X-C chemokine receptor type 4 (CXCR4) antagonist plerixafor) to counteract protective resistance mechanisms.

The development of a technology-integrated precision medicine system has emerged as a promising direction based on current research progress, with its core framework focusing on establishing a functional precision medicine platform [188]. This platform seeks to integrate multi-omics data via intelligent diagnostic systems while incorporating emerging technologies including epigenetic editing and metabolic reprogramming to investigate dynamic treatment optimization. Such a system would prioritize phenotype-specific targeted therapeutic strategies and potentially accelerate the development of breakthrough therapies such as immunogenomics-based approaches. The gradual implementation of personalized treatment algorithms may create new opportunities for achieving more sustained remission in AML patients. This research paradigm—connecting mechanistic understanding, technological integration, and clinical translation—could represent a critical transition in AML treatment evolution from empirical approaches to precision medicine.

## Figures and Tables

**Table 1:** Several FDA-approved targeted therapies for AML.

Drug	Target	Date of Approval	Monotherapy	Combination	Patient Types
Midostaurin	FLT3	28 April 2017		Standard cytarabine + daunorubicin induction + cytarabine consolidation	Newly diagnosed AML that is *FLT3*-mutation-positive
Gilteritinib	FLT3	28 November 2018	√		Adult patients who have relapsed or refractory AML with an *FLT3* mutation
Quizartinib	FLT3	20 July 2023		Standard cytarabine + anthracycline induction + cytarabine consolidation	Adult patients with newly diagnosed AML that is *FLT3* ITD-positive
Ivosidenib	IDH1	20 July 2018	√		Adult patients with relapsed or refractory AML with a susceptible *IDH1* mutation
		2 May 2019	√		Adult patients with newly diagnosed AML who are ≥75 years old or who have comorbidities that preclude the use of intensive induction chemotherapy
Olutasidenib	IDH1	1 December 2022	√		Adult patients with relapsed or refractory AML with a susceptible *IDH1* mutation
Enasidenib	IDH2	1 August 2017	√		Adult patients with relapsed or refractory AML with an *IDH2* mutation
Venetoclax	BCL2	21 November 2018		Azacitidine or decitabine or low-dose cytarabine	Newly diagnosed AML in adults who are age 75 years or older, or who have comorbidities that preclude the use of intensive induction chemotherapy
Gemtuzumab Ozogamicin	CD33	1 September 2017	√		Newly diagnosed CD33-positive AML in adults Relapsed or refractory CD33-positive AML in adults and in pediatric patients 2 years and older
				Daunorubicin + cytarabine	Newly diagnosed, de novo AML
		16 June 2020	√		Newly diagnosed CD33-positive AML in adults and pediatric patients 1 month and older
Glasdegib	Hedgehog pathway	21 November 2018		Low-dose cytarabine	Adult patients who are ≥75 years old or who have comorbidities that preclude the use of intensive induction chemotherapy
Revumenib	Menin	15 November 2024	√		Relapsed or refractory acute leukemia with a *KMT2A* gene translocation in adult and pediatric patients 1 year and older

Abbreviations: *FLT3*, FMS-like tyrosine kinase 3; ITD, internal tandem duplication; *IDH1*, isocitrate dehydrogenase 1; *IDH2*, isocitrate dehydrogenase 2; *KMT2A*, lysine methyltransferase 2A.

**Table 2:** Mutations inducing chemotherapy resistance.

Mutation	Resistance Mechanisms	Clinical Impact
*TP53*	Disrupts DNA damage repair and apoptosis pathways. Induces genomic instability, accelerating resistant clone expansion. Synergizes with *NRAS* to promote inflammation via GATA2 downregulation.	Poor prognosis marker. Associated with complex karyotypes. Worse outcomes in elderly patients.
*RAS*	Mutant clones selected under chemotherapy pressure. Synergizes with *TP53* to induce inflammation and chemoresistance.	Higher activation frequency in elderly AML patients. Contributes to poor outcomes.
*MLL*	Fusion proteins (e.g., MLL-AF9) activate *HOXA9*/*MEIS1* via DOT1L-mediated H3K79 hypermethylation. Upregulates EMT-related genes (*SNAIL*, *TWIST*), enhancing invasiveness.	Prevalent in ~23% of t-AML. Drives relapse through survival pathway activation.
*FLT3* *-ITD*	Drives clonal expansion with increased allele burden at relapse. Persists post-chemotherapy, dominating relapse.	Independent prognostic factor for chemotherapy failure. Strongly associated with poor survival.
*IDH1/2*	Neomorphic production of (R)-2-HG inhibits α-KG-dependent enzymes (TET2, Jumonji demethylases), causing epigenetic dysregulation. Promotes leukemic stem cell survival.	Early acquisition and stable retention during progression. Drives relapse and chemoresistance.
*DNMT3A*	Causes global hypomethylation and focal promoter hypermethylation. *R882H* mutation activates NRF2/NQO1 pathway, reducing daunorubicin sensitivity. Synergizes with *NPM1c*/*FLT3-ITD* to upregulate anti-apoptotic genes and disrupt chromatin remodeling. Promotes TWIST1 expression, facilitating extramedullary infiltration.	Higher relapse rates and reduced OS. *R882H* associated with poor outcomes in younger patients.
*ASXL1*	Truncation mutations disrupt PRC2-mediated H3K27 methylation, sustaining LSCs in an undifferentiated state. Synergizes with CEBPA mutations to suppress ribosome biogenesis, DNA damage response, and immune activation.	Associated with poor prognosis and chemoresistance. Accelerates AML progression in synergy with other mutations.

## References

[1] Döhner H., Estey E., Grimwade D., Amadori S., Appelbaum F.R., Büchner T., Dombret H., Ebert B.L., Fenaux P., Larson R.A. (2017). Diagnosis and Management of AML in Adults: 2017 ELN Recommendations from an International Expert Panel. Blood.

[2] Döhner H., Estey E.H., Amadori S., Appelbaum F.R., Büchner T., Burnett A.K., Dombret H., Fenaux P., Grimwade D., Larson R.A. (2010). Diagnosis and Management of Acute Myeloid Leukemia in Adults: Recommendations from an International Expert Panel, on Behalf of the European LeukemiaNet. Blood.

[3] DiNardo C.D., Erba H.P., Freeman S.D., Wei A.H. (2023). Acute Myeloid Leukaemia. Lancet.

[4] Kantarjian H., Kadia T., DiNardo C., Daver N., Borthakur G., Jabbour E., Garcia-Manero G., Konopleva M., Ravandi F. (2021). Acute Myeloid Leukemia: Current Progress and Future Directions. Blood Cancer J..

[5] Bhansali R.S., Pratz K.W., Lai C. (2023). Recent Advances in Targeted Therapies in Acute Myeloid Leukemia. J. Hematol. Oncol..

[6] Krauss A.C., Gao X., Li L., Manning M.L., Patel P., Fu W., Janoria K.G., Gieser G., Bateman D.A., Przepiorka D. (2019). FDA Approval Summary: (Daunorubicin and Cytarabine) Liposome for Injection for the Treatment of Adults with High-Risk Acute Myeloid Leukemia. Clin. Cancer Res..

[7] Appelbaum F.R., Gundacker H., Head D.R., Slovak M.L., Willman C.L., Godwin J.E., Anderson J.E., Petersdorf S.H. (2006). Age and Acute Myeloid Leukemia. Blood.

[8] Tardi P., Johnstone S., Harasym N., Xie S., Harasym T., Zisman N., Harvie P., Bermudes D., Mayer L. (2009). In Vivo Maintenance of Synergistic Cytarabine:Daunorubicin Ratios Greatly Enhances Therapeutic Efficacy. Leuk. Res..

[9] Lancet J.E., Uy G.L., Cortes J.E., Newell L.F., Lin T.L., Ritchie E.K., Stuart R.K., Strickland S.A., Hogge D., Solomon S.R. (2018). CPX-351 (Cytarabine and Daunorubicin) Liposome for Injection Versus Conventional Cytarabine Plus Daunorubicin in Older Patients with Newly Diagnosed Secondary Acute Myeloid Leukemia. J. Clin. Oncol..

[10] Kantarjian H.M., DiNardo C.D., Kadia T.M., Daver N.G., Altman J.K., Stein E.M., Jabbour E., Schiffer C.A., Lang A., Ravandi F. (2025). Acute Myeloid Leukemia Management and Research in 2025. CA Cancer J. Clin..

[11] Short N.J., Kantarjian H. (2021). When Less Is More: Reevaluating the Role of Intensive Chemotherapy for Older Adults with Acute Myeloid Leukemia in the Modern Era. J. Clin. Oncol..

[12] Kantarjian H., Short N.J., DiNardo C., Stein E.M., Daver N., Perl A.E., Wang E.S., Wei A., Tallman M. (2021). Harnessing the Benefits of Available Targeted Therapies in Acute Myeloid Leukaemia. Lancet Haematol..

[13] DiNardo C.D., Jonas B.A., Pullarkat V., Thirman M.J., Garcia J.S., Wei A.H., Konopleva M., Döhner H., Letai A., Fenaux P. (2020). Azacitidine and Venetoclax in Previously Untreated Acute Myeloid Leukemia. N. Engl. J. Med..

[14] Lim Z.-F., Ma P.C. (2019). Emerging Insights of Tumor Heterogeneity and Drug Resistance Mechanisms in Lung Cancer Targeted Therapy. J. Hematol. Oncol..

[15] O’Reilly E., Zeinabad H.A., Szegezdi E. (2021). Hematopoietic versus Leukemic Stem Cell Quiescence: Challenges and Therapeutic Opportunities. Blood Rev..

[16] Li S., Garrett-Bakelman F.E., Chung S.S., Sanders M.A., Hricik T., Rapaport F., Patel J., Dillon R., Vijay P., Brown A.L. (2016). Distinct Evolution and Dynamics of Epigenetic and Genetic Heterogeneity in Acute Myeloid Leukemia. Nat. Med..

[17] Ma X., Liu Y., Liu Y., Alexandrov L.B., Edmonson M.N., Gawad C., Zhou X., Li Y., Rusch M.C., Easton J. (2018). Pan-Cancer Genome and Transcriptome Analyses of 1,699 Paediatric Leukaemias and Solid Tumours. Nature.

[18] Takahashi K., Tanaka T. (2023). Clonal Evolution and Hierarchy in Myeloid Malignancies. Trends Cancer.

[19] Parkin B., Ouillette P., Li Y., Keller J., Lam C., Roulston D., Li C., Shedden K., Malek S.N. (2013). Clonal Evolution and Devolution after Chemotherapy in Adult Acute Myelogenous Leukemia. Blood.

[20] Ding L., Ley T.J., Larson D.E., Miller C.A., Koboldt D.C., Welch J.S., Ritchey J.K., Young M.A., Lamprecht T., McLellan M.D. (2012). Clonal Evolution in Relapsed Acute Myeloid Leukaemia Revealed by Whole-Genome Sequencing. Nature.

[21] Shlush L.I., Mitchell A., Heisler L., Abelson S., Ng S.W.K., Trotman-Grant A., Medeiros J.J.F., Rao-Bhatia A., Jaciw-Zurakowsky I., Marke R. (2017). Tracing the Origins of Relapse in Acute Myeloid Leukaemia to Stem Cells. Nature.

[22] Wong T.N., Ramsingh G., Young A.L., Miller C.A., Touma W., Welch J.S., Lamprecht T.L., Shen D., Hundal J., Fulton R.S. (2015). Role of TP53 Mutations in the Origin and Evolution of Therapy-Related Acute Myeloid Leukaemia. Nature.

[23] Döhner H., Wei A.H., Appelbaum F.R., Craddock C., DiNardo C.D., Dombret H., Ebert B.L., Fenaux P., Godley L.A., Hasserjian R.P. (2022). Diagnosis and Management of AML in Adults: 2022 Recommendations from an International Expert Panel on Behalf of the ELN. Blood.

[24] Itzykson R., Fournier E., Berthon C., Röllig C., Braun T., Marceau-Renaut A., Pautas C., Nibourel O., Lemasle E., Micol J.-B. (2021). Genetic Identification of Patients with AML Older than 60 Years Achieving Long-Term Survival with Intensive Chemotherapy. Blood.

[25] Rücker F.G., Schlenk R.F., Bullinger L., Kayser S., Teleanu V., Kett H., Habdank M., Kugler C.-M., Holzmann K., Gaidzik V.I. (2012). TP53 Alterations in Acute Myeloid Leukemia with Complex Karyotype Correlate with Specific Copy Number Alterations, Monosomal Karyotype, and Dismal Outcome. Blood.

[26] Rajagopalan A., Feng Y., Gayatri M.B., Ranheim E.A., Klungness T., Matson D.R., Lee M.H., Jung M.M., Zhou Y., Gao X. (2003). A Gain-of-Function P53 Mutant Synergizes with Oncogenic NRAS to Promote Acute Myeloid Leukemia in Mice. J. Clin. Investig..

[27] Rao A.V., Valk P.J.M., Metzeler K.H., Acharya C.R., Tuchman S.A., Stevenson M.M., Rizzieri D.A., Delwel R., Buske C., Bohlander S.K. (2009). Age-Specific Differences in Oncogenic Pathway Dysregulation in Patients with Acute Myeloid Leukemia. J. Clin. Oncol..

[28] Faber J., Krivtsov A.V., Stubbs M.C., Wright R., Davis T.N., van den Heuvel-Eibrink M., Zwaan C.M., Kung A.L., Armstrong S.A. (2009). HOXA9 Is Required for Survival in Human MLL-Rearranged Acute Leukemias. Blood.

[29] Li Z., Chen P., Su R., Hu C., Li Y., Elkahloun A.G., Zuo Z., Gurbuxani S., Arnovitz S., Weng H. (2016). PBX3 and MEIS1 Cooperate in Hematopoietic Cells to Drive Acute Myeloid Leukemias Characterized by a Core Transcriptome of the MLL-Rearranged Disease. Cancer Res..

[30] Stavropoulou V., Kaspar S., Brault L., Sanders M.A., Juge S., Morettini S., Tzankov A., Iacovino M., Lau I.-J., Milne T.A. (2016). MLL-AF9 Expression in Hematopoietic Stem Cells Drives a Highly Invasive AML Expressing EMT-Related Genes Linked to Poor Outcome. Cancer Cell.

[31] Daver N., Schlenk R.F., Russell N.H., Levis M.J. (2019). Targeting FLT3 Mutations in AML: Review of Current Knowledge and Evidence. Leukemia.

[32] Angenendt L., Röllig C., Montesinos P., Martínez-Cuadrón D., Barragan E., García R., Botella C., Martínez P., Ravandi F., Kadia T. (2019). Chromosomal Abnormalities and Prognosis in NPM1-Mutated Acute Myeloid Leukemia: A Pooled Analysis of Individual Patient Data From Nine International Cohorts. J. Clin. Oncol..

[33] Krönke J., Bullinger L., Teleanu V., Tschürtz F., Gaidzik V.I., Kühn M.W.M., Rücker F.G., Holzmann K., Paschka P., Kapp-Schwörer S. (2013). Clonal Evolution in Relapsed NPM1-Mutated Acute Myeloid Leukemia. Blood.

[34] Xu W., Yang H., Liu Y., Yang Y., Wang P., Kim S.-H., Ito S., Yang C., Wang P., Xiao M.-T. (2011). Oncometabolite 2-Hydroxyglutarate Is a Competitive Inhibitor of α-Ketoglutarate-Dependent Dioxygenases. Cancer Cell.

[35] Chan S.M., Thomas D., Corces-Zimmerman M.R., Xavy S., Rastogi S., Hong W.-J., Zhao F., Medeiros B.C., Tyvoll D.A., Majeti R. (2015). Isocitrate Dehydrogenase 1 and 2 Mutations Induce BCL-2 Dependence in Acute Myeloid Leukemia. Nat. Med..

[36] Im A.P., Sehgal A.R., Carroll M.P., Smith B.D., Tefferi A., Johnson D.E., Boyiadzis M. (2014). DNMT3A and IDH Mutations in Acute Myeloid Leukemia and Other Myeloid Malignancies: Associations with Prognosis and Potential Treatment Strategies. Leukemia.

[37] Shlush L.I., Zandi S., Mitchell A., Chen W.C., Brandwein J.M., Gupta V., Kennedy J.A., Schimmer A.D., Schuh A.C., Yee K.W. (2014). Identification of Pre-Leukaemic Haematopoietic Stem Cells in Acute Leukaemia. Nature.

[38] Xu J., Zhang W., Yan X.-J., Lin X.-Q., Li W., Mi J.-Q., Li J.-M., Zhu J., Chen Z., Chen S.-J. (2016). DNMT3A Mutation Leads to Leukemic Extramedullary Infiltration Mediated by TWIST1. J. Hematol. Oncol..

[39] Chu X., Zhong L., Dan W., Wang X., Zhang Z., Liu Z., Lu Y., Shao X., Zhou Z., Chen S. (2022). DNMT3A R882H Mutation Drives Daunorubicin Resistance in Acute Myeloid Leukemia via Regulating NRF2/NQO1 Pathway. Cell Commun. Signal..

[40] Guryanova O.A., Shank K., Spitzer B., Luciani L., Koche R.P., Garrett-Bakelman F.E., Ganzel C., Durham B.H., Mohanty A., Hoermann G. (2016). DNMT3A R882 Mutations Promote Anthracycline Resistance in Acute Myeloid Leukemia through Impaired Nucleosome Remodeling. Nat. Med..

[41] Marcucci G., Metzeler K.H., Schwind S., Becker H., Maharry K., Mrózek K., Radmacher M.D., Kohlschmidt J., Nicolet D., Whitman S.P. (2012). Age-Related Prognostic Impact of Different Types of DNMT3A Mutations in Adults with Primary Cytogenetically Normal Acute Myeloid Leukemia. J. Clin. Oncol..

[42] D’Altri T., Wilhelmson A.S., Schuster M.B., Wenzel A., Kalvisa A., Pundhir S., Hansen A.M., Porse B.T. (2021). The ASXL1-G643W Variant Accelerates the Development of CEBPA Mutant Acute Myeloid Leukemia. Haematologica.

[43] Inoue D., Matsumoto M., Nagase R., Saika M., Fujino T., Nakayama K.I., Kitamura T. (2016). Truncation Mutants of ASXL1 Observed in Myeloid Malignancies Are Expressed at Detectable Protein Levels. Exp. Hematol..

[44] Ferrando A.A., López-Otín C. (2017). Clonal Evolution in Leukemia. Nat. Med..

[45] Thomas D., Majeti R. (2017). Biology and Relevance of Human Acute Myeloid Leukemia Stem Cells. Blood.

[46] Ho T.-C., LaMere M., Stevens B.M., Ashton J.M., Myers J.R., O’Dwyer K.M., Liesveld J.L., Mendler J.H., Guzman M., Morrissette J.D. (2016). Evolution of Acute Myelogenous Leukemia Stem Cell Properties after Treatment and Progression. Blood.

[47] Ng S.W.K., Mitchell A., Kennedy J.A., Chen W.C., McLeod J., Ibrahimova N., Arruda A., Popescu A., Gupta V., Schimmer A.D. (2016). A 17-Gene Stemness Score for Rapid Determination of Risk in Acute Leukaemia. Nature.

[48] Saito Y., Kitamura H., Hijikata A., Tomizawa-Murasawa M., Tanaka S., Takagi S., Uchida N., Suzuki N., Sone A., Najima Y. (2010). Identification of Therapeutic Targets for Quiescent, Chemotherapy-Resistant Human Leukemia Stem Cells. Sci. Transl. Med..

[49] Sasca D., Szybinski J., Schüler A., Shah V., Heidelberger J., Haehnel P.S., Dolnik A., Kriege O., Fehr E.-M., Gebhardt W.H. (2019). NCAM1 (CD56) Promotes Leukemogenesis and Confers Drug Resistance in AML. Blood.

[50] Williams M.S., Amaral F.M., Simeoni F., Somervaille T.C. (2020). A Stress-Responsive Enhancer Induces Dynamic Drug Resistance in Acute Myeloid Leukemia. J. Clin. Investig..

[51] Wulf G.G., Wang R.-Y., Kuehnle I., Weidner D., Marini F., Brenner M.K., Andreeff M., Goodell M.A. (2001). A Leukemic Stem Cell with Intrinsic Drug Efflux Capacity in Acute Myeloid Leukemia. Blood.

[52] Ceraulo A., Lapillonne H., Cheok M.H., Preudhomme C., Dombret H., Terré C., Lambert J., Leverger G., Bertrand Y., Mortreux F. (2022). Prognostic Impact of ABCA3 Expression in Adult and Pediatric Acute Myeloid Leukemia: An ALFA-ELAM02 Joint Study. Blood Adv..

[53] Steinbach D., Gillet J.-P., Sauerbrey A., Gruhn B., Dawczynski K., Bertholet V., de Longueville F., Zintl F., Remacle J., Efferth T. (2006). ABCA3 as a Possible Cause of Drug Resistance in Childhood Acute Myeloid Leukemia. Clin. Cancer Res..

[54] Shaffer B.C., Gillet J.-P., Patel C., Baer M.R., Bates S.E., Gottesman M.M. (2012). Drug Resistance: Still a Daunting Challenge to the Successful Treatment of AML. Drug Resist. Updates.

[55] Saadatpour A., Guo G., Orkin S.H., Yuan G.-C. (2014). Characterizing Heterogeneity in Leukemic Cells Using Single-Cell Gene Expression Analysis. Genome Biol..

[56] Lagadinou E.D., Sach A., Callahan K., Rossi R.M., Neering S.J., Minhajuddin M., Ashton J.M., Pei S., Grose V., O’Dwyer K.M. (2013). BCL-2 Inhibition Targets Oxidative Phosphorylation and Selectively Eradicates Quiescent Human Leukemia Stem Cells. Cell Stem Cell.

[57] Jones C.L., Stevens B.M., D’Alessandro A., Reisz J.A., Culp-Hill R., Nemkov T., Pei S., Khan N., Adane B., Ye H. (2018). Inhibition of Amino Acid Metabolism Selectively Targets Human Leukemia Stem Cells. Cancer Cell.

[58] Pei S., Minhajuddin M., Adane B., Khan N., Stevens B.M., Mack S.C., Lai S., Rich J.N., Inguva A., Shannon K.M. (2018). AMPK/FIS1-Mediated Mitophagy Is Required for Self-Renewal of Human AML Stem Cells. Cell Stem Cell.

[59] Farge T., Saland E., De Toni F., Aroua N., Hosseini M., Perry R., Bosc C., Sugita M., Stuani L., Fraisse M. (2017). Chemotherapy-Resistant Human Acute Myeloid Leukemia Cells Are Not Enriched for Leukemic Stem Cells but Require Oxidative Metabolism. Cancer Discov..

[60] Hassan N., Yi H., Malik B., Gaspard-Boulinc L., Samaraweera S.E., Casolari D.A., Seneviratne J., Balachandran A., Chew T., Duly A. (2024). Loss of the Stress Sensor GADD45A Promotes Stem Cell Activity and Ferroptosis Resistance in LGR4/HOXA9-Dependent AML. Blood.

[61] Yang X., Liu J., Liu W., Wu H., Wei Y., Guo X., Jia H., Can C., Wang D., Hu X. (2024). circFAM193B Interaction with PRMT6 Regulates AML Leukemia Stem Cells Chemoresistance through Altering the Oxidative Metabolism and Lipid Peroxidation. Leukemia.

[62] Tian Y., Huang Z., Wang Z., Yin C., Zhou L., Zhang L., Huang K., Zhou H., Jiang X., Li J. (2014). Identification of Novel Molecular Markers for Prognosis Estimation of Acute Myeloid Leukemia: Over-Expression of PDCD7, FIS1 and Ang2 May Indicate Poor Prognosis in Pretreatment Patients with Acute Myeloid Leukemia. PLoS ONE.

[63] Wang X., Huang S., Chen J.-L. (2017). Understanding of Leukemic Stem Cells and Their Clinical Implications. Mol. Cancer.

[64] Lechman E.R., Gentner B., Ng S.W., Schoof E.M., van Galen P., Kennedy J.A., Nucera S., Ciceri F., Kaufmann K.B., Takayama N. (2016). miR-126 Regulates Distinct Self-Renewal Outcomes in Normal and Malignant Hematopoietic Stem Cells. Cancer Cell.

[65] Gu H., Chen C., Hou Z.-S., He X.-D., Xie S., Ni J., Qian C., Cheng X., Jiang T., Yang C. (2024). PI3Kγ Maintains the Self-Renewal of Acute Myeloid Leukemia Stem Cells by Regulating the Pentose Phosphate Pathway. Blood.

[66] Carter J.L., Hege K., Yang J., Kalpage H.A., Su Y., Edwards H., Hüttemann M., Taub J.W., Ge Y. (2020). Targeting Multiple Signaling Pathways: The New Approach to Acute Myeloid Leukemia Therapy. Signal Transduct. Target. Ther..

[67] Freisleben F., Behrmann L., Thaden V., Muschhammer J., Bokemeyer C., Fiedler W., Wellbrock J. (2020). Downregulation of GLI3 Expression Mediates Chemotherapy Resistance in Acute Myeloid Leukemia. Int. J. Mol. Sci..

[68] Lane S.W., Wang Y.J., Celso C.L., Ragu C., Bullinger L., Sykes S.M., Ferraro F., Shterental S., Lin C.P., Gilliland D.G. (2011). Differential Niche and Wnt Requirements during Acute Myeloid Leukemia Progression. Blood.

[69] Méndez-Ferrer S., Bonnet D., Steensma D.P., Hasserjian R.P., Ghobrial I.M., Gribben J.G., Andreeff M., Krause D.S. (2020). Bone Marrow Niches in Haematological Malignancies. Nat. Rev. Cancer.

[70] Zhou H.S., Carter B.Z., Andreeff M. (2016). Bone Marrow Niche-Mediated Survival of Leukemia Stem Cells in Acute Myeloid Leukemia: Yin and Yang. Cancer Biol. Med..

[71] Konopleva M.Y., Jordan C.T. (2011). Leukemia Stem Cells and Microenvironment: Biology and Therapeutic Targeting. J. Clin. Oncol..

[72] Fei M.-Y., Wang Y., Chang B.-H., Xue K., Dong F., Huang D., Li X.-Y., Li Z.-J., Hu C.-L., Liu P. (2023). The Non-Cell-Autonomous Function of ID1 Promotes AML Progression via ANGPTL7 from the Microenvironment. Blood.

[73] Welner R.S., Amabile G., Bararia D., Czibere A., Yang H., Zhang H., Pontes L.L.D.F., Ye M., Levantini E., Di Ruscio A. (2015). Treatment of Chronic Myelogenous Leukemia by Blocking Cytokine Alterations Found in Normal Stem and Progenitor Cells. Cancer Cell.

[74] Kleppe M., Kwak M., Koppikar P., Riester M., Keller M., Bastian L., Hricik T., Bhagwat N., McKenney A.S., Papalexi E. (2015). JAK–STAT Pathway Activation in Malignant and Nonmalignant Cells Contributes to MPN Pathogenesis and Therapeutic Response. Cancer Discov..

[75] Flores-Figueroa E., Montesinos J.J., Flores-Guzmán P., Gutiérrez-Espíndola G., Arana-Trejo R.M., Castillo-Medina S., Pérez-Cabrera A., Hernández-Estévez E., Arriaga L., Mayani H. (2008). Functional Analysis of Myelodysplastic Syndromes-Derived Mesenchymal Stem Cells. Leuk. Res..

[76] Medyouf H., Mossner M., Jann J.-C., Nolte F., Raffel S., Herrmann C., Lier A., Eisen C., Nowak V., Zens B. (2014). Myelodysplastic Cells in Patients Reprogram Mesenchymal Stromal Cells to Establish a Transplantable Stem Cell Niche Disease Unit. Cell Stem Cell.

[77] Jacamo R., Chen Y., Wang Z., Ma W., Zhang M., Spaeth E.L., Wang Y., Battula V.L., Mak P.Y., Schallmoser K. (2014). Reciprocal Leukemia-Stroma VCAM-1/VLA-4-Dependent Activation of NF-κB Mediates Chemoresistance. Blood.

[78] Layani-Bazar A., Skornick I., Berrebi A., Pauker M.H., Noy E., Silberman A., Albeck M., Longo D.L., Kalechman Y., Sredni B. (2014). Redox Modulation of Adjacent Thiols in VLA-4 by AS101 Converts Myeloid Leukemia Cells from a Drug-Resistant to Drug-Sensitive State. Cancer Res..

[79] Matsunaga T., Takemoto N., Sato T., Takimoto R., Tanaka I., Fujimi A., Akiyama T., Kuroda H., Kawano Y., Kobune M. (2003). Interaction between Leukemic-Cell VLA-4 and Stromal Fibronectin Is a Decisive Factor for Minimal Residual Disease of Acute Myelogenous Leukemia. Nat. Med..

[80] Borella G., Da Ros A., Borile G., Porcù E., Tregnago C., Benetton M., Marchetti A., Bisio V., Montini B., Michielotto B. (2021). Targeting the Plasticity of Mesenchymal Stromal Cells to Reroute the Course of Acute Myeloid Leukemia. Blood.

[81] Xiao P., Sandhow L., Heshmati Y., Kondo M., Bouderlique T., Dolinska M., Johansson A.-S., Sigvardsson M., Ekblom M., Walfridsson J. (2018). Distinct Roles of Mesenchymal Stem and Progenitor Cells during the Development of Acute Myeloid Leukemia in Mice. Blood Adv..

[82] Cai H., Kondo M., Sandhow L., Xiao P., Johansson A.-S., Sasaki T., Zawacka-Pankau J., Tryggvason K., Ungerstedt J., Walfridsson J. (2022). Critical Role of *Lama4* for Hematopoiesis Regeneration and Acute Myeloid Leukemia Progression. Blood.

[83] Zhang J., Niu C., Ye L., Huang H., He X., Tong W.-G., Ross J., Haug J., Johnson T., Feng J.Q. (2003). Identification of the Haematopoietic Stem Cell Niche and Control of the Niche Size. Nature.

[84] Kode A., Manavalan J.S., Mosialou I., Bhagat G., Rathinam C.V., Luo N., Khiabanian H., Lee A., Murty V.V., Friedman R. (2014). Leukaemogenesis Induced by an Activating β-Catenin Mutation in Osteoblasts. Nature.

[85] Liersch R., Gerss J., Schliemann C., Bayer M., Schwöppe C., Biermann C., Appelmann I., Kessler T., Löwenberg B., Büchner T. (2012). Osteopontin Is a Prognostic Factor for Survival of Acute Myeloid Leukemia Patients. Blood.

[86] Galán-Díez M., Borot F., Ali A.M., Zhao J., Gil-Iturbe E., Shan X., Luo N., Liu Y., Huang X.-P., Bisikirska B. (2022). Subversion of Serotonin Receptor Signaling in Osteoblasts by Kynurenine Drives Acute Myeloid Leukemia. Cancer Discov..

[87] Prendergast G.C., Malachowski W.P., DuHadaway J.B., Muller A.J. (2017). Discovery of IDO1 Inhibitors: From Bench to Bedside. Cancer Res..

[88] Krevvata M., Silva B.C., Manavalan J.S., Galan-Diez M., Kode A., Matthews B.G., Park D., Zhang C.A., Galili N., Nickolas T.L. (2014). Inhibition of Leukemia Cell Engraftment and Disease Progression in Mice by Osteoblasts. Blood.

[89] Xie X., Zhang W., Xiao M., Wei T., Qiu Y., Qiu J., Wang H., Qiu Z., Zhang S., Pan Y. (2023). TREM2 Acts as a Receptor for IL-34 to Suppress Acute Myeloid Leukemia in Mice. Blood.

[90] Candoni A., Papayannidis C., Martinelli G., Simeone E., Gottardi M., Iacobucci I., Gherlinzoni F., Visani G., Baccarani M., Fanin R. (2018). Flai (Fludarabine, Cytarabine, Idarubicin) plus Low-Dose Gemtuzumab Ozogamicin as Induction Therapy in CD33-Positive AML: Final Results and Long Term Outcome of a Phase II Multicenter Clinical Trial. Am. J. Hematol..

[91] Montillo M., Mirto S., Petti M.C., Latagliata R., Magrin S., Pinto A., Zagonel V., Mele G., Tedeschi A., Ferrara F. (1998). Fludarabine, Cytarabine, and G-CSF (FLAG) for the Treatment of Poor Risk Acute Myeloid Leukemia. Am. J. Hematol..

[92] Burnett A.K., Russell N.H., Hills R.K., Hunter A.E., Kjeldsen L., Yin J., Gibson B.E., Wheatley K., Milligan D. (2013). Optimization of Chemotherapy for Younger Patients with Acute Myeloid Leukemia: Results of the Medical Research Council AML15 Trial. J. Clin. Oncol..

[93] Borthakur G., Ravandi F., Patel K., Wang X., Kadia T., DiNardo C., Garcia-Manero G., Pemmaraju N., Jabbour E.J., Takahashi K. (2022). Retrospective Comparison of Survival and Responses to Fludarabine, Cytarabine, GCSF (FLAG) in Combination with Gemtuzumab Ozogamicin (GO) or Idarubicin (IDA) in Patients with Newly Diagnosed Core Binding Factor (CBF) Acute Myelogenous Leukemia: MD Anderson Experience in 174 Patients. Am. J. Hematol..

[94] Holowiecki J., Grosicki S., Giebel S., Robak T., Kyrcz-Krzemien S., Kuliczkowski K., Skotnicki A.B., Hellmann A., Sulek K., Dmoszynska A. (2012). Cladribine, But Not Fludarabine, Added to Daunorubicin and Cytarabine During Induction Prolongs Survival of Patients with Acute Myeloid Leukemia: A Multicenter, Randomized Phase III Study. J. Clin. Oncol..

[95] Yates J.W., Wallace H.J., Ellison R.R., Holland J.F. (1973). Cytosine Arabinoside (NSC-63878) and Daunorubicin (NSC-83142) Therapy in Acute Nonlymphocytic Leukemia. Cancer Chemother. Rep..

[96] Kantarjian H.M., Thomas X.G., Dmoszynska A., Wierzbowska A., Mazur G., Mayer J., Gau J.-P., Chou W.-C., Buckstein R., Cermak J. (2012). Multicenter, Randomized, Open-Label, Phase III Trial of Decitabine versus Patient Choice, with Physician Advice, of Either Supportive Care or Low-Dose Cytarabine for the Treatment of Older Patients with Newly Diagnosed Acute Myeloid Leukemia. J. Clin. Oncol..

[97] Iwai T., Yokota S., Nakao M., Okamoto T., Taniwaki M., Onodera N., Watanabe A., Kikuta A., Tanaka A., Asami K. (1999). Internal Tandem Duplication of the FLT3 Gene and Clinical Evaluation in Childhood Acute Myeloid Leukemia. Leukemia.

[98] Macečková D., Vaňková L., Holubová M., Jindra P., Klieber R., Jandová E., Pitule P. (2024). Current Knowledge about FLT3 Gene Mutations, Exploring the Isoforms, and Protein Importance in AML. Mol. Biol. Rep..

[99] Stone R.M., Fischer T., Paquette R., Schiller G., Schiffer C.A., Ehninger G., Cortes J., Kantarjian H.M., DeAngelo D.J., Huntsman-Labed A. (2012). Phase IB Study of the FLT3 Kinase Inhibitor Midostaurin with Chemotherapy in Younger Newly Diagnosed Adult Patients with Acute Myeloid Leukemia. Leukemia.

[100] Stone R.M., Mandrekar S.J., Sanford B.L., Laumann K., Geyer S., Bloomfield C.D., Thiede C., Prior T.W., Döhner K., Marcucci G. (2017). Midostaurin plus Chemotherapy for Acute Myeloid Leukemia with a FLT3 Mutation. N. Engl. J. Med..

[101] Von Bubnoff N., Engh R.A., Åberg E., Sänger J., Peschel C., Duyster J. (2009). FMS-Like Tyrosine Kinase 3–Internal Tandem Duplication Tyrosine Kinase Inhibitors Display a Nonoverlapping Profile of Resistance Mutations In Vitro. Cancer Res..

[102] Heidel F., Solem F.K., Breitenbuecher F., Lipka D.B., Kasper S., Thiede M.H., Brandts C., Serve H., Roesel J., Giles F. (2006). Clinical Resistance to the Kinase Inhibitor PKC412 in Acute Myeloid Leukemia by Mutation of Asn-676 in the FLT3 Tyrosine Kinase Domain. Blood.

[103] Rummelt C., Gorantla S.P., Meggendorfer M., Charlet A., Endres C., Döhner K., Heidel F.H., Fischer T., Haferlach T., Duyster J. (2021). Activating JAK-Mutations Confer Resistance to FLT3 Kinase Inhibitors in FLT3-ITD Positive AML in Vitro and in Vivo. Leukemia.

[104] Schmalbrock L.K., Dolnik A., Cocciardi S., Sträng E., Theis F., Jahn N., Panina E., Blätte T.J., Herzig J., Skambraks S. (2021). Clonal Evolution of Acute Myeloid Leukemia with FLT3-ITD Mutation under Treatment with Midostaurin. Blood.

[105] Döhner H., Wei A.H., Löwenberg B. (2021). Towards Precision Medicine for AML. Nat. Rev. Clin. Oncol..

[106] Perl A.E., Martinelli G., Cortes J.E., Neubauer A., Berman E., Paolini S., Montesinos P., Baer M.R., Larson R.A., Ustun C. (2019). Gilteritinib or Chemotherapy for Relapsed or Refractory FLT3-Mutated AML. N. Engl. J. Med..

[107] Joshi S.K., Nechiporuk T., Bottomly D., Piehowski P.D., Reisz J.A., Pittsenbarger J., Kaempf A., Gosline S.J., Wang Y.-T., Hansen J.R. (2021). The AML Microenvironment Catalyzes a Stepwise Evolution to Gilteritinib Resistance. Cancer Cell.

[108] McMahon C.M., Ferng T., Canaani J., Wang E.S., Morrissette J.J., Eastburn D.J., Pellegrino M., Durruthy-Durruthy R., Watt C.D., Asthana S. (2019). Clonal Selection with RAS Pathway Activation Mediates Secondary Clinical Resistance to Selective FLT3 Inhibition in Acute Myeloid Leukemia. Cancer Discov..

[109] Sabatier M., Birsen R., Lauture L., Mouche S., Angelino P., Dehairs J., Goupille L., Boussaid I., Heiblig M., Boet E. (2023). C/EBPα Confers Dependence to Fatty Acid Anabolic Pathways and Vulnerability to Lipid Oxidative Stress-Induced Ferroptosis in FLT3-Mutant Leukemia. Cancer Discov..

[110] Smith C.C., Wang Q., Chin C.-S., Salerno S., Damon L.E., Levis M.J., Perl A.E., Travers K.J., Wang S., Hunt J.P. (2012). Validation of ITD Mutations in FLT3 as a Therapeutic Target in Human Acute Myeloid Leukaemia. Nature.

[111] Smith C.C., Paguirigan A., Jeschke G.R., Lin K.C., Massi E., Tarver T., Chin C.-S., Asthana S., Olshen A., Travers K.J. (2017). Heterogeneous Resistance to Quizartinib in Acute Myeloid Leukemia Revealed by Single-Cell Analysis. Blood.

[112] Göllner S., Oellerich T., Agrawal-Singh S., Schenk T., Klein H.-U., Rohde C., Pabst C., Sauer T., Lerdrup M., Tavor S. (2017). Loss of the Histone Methyltransferase EZH2 Induces Resistance to Multiple Drugs in Acute Myeloid Leukemia. Nat. Med..

[113] Gebru M.T., Wang H.-G. (2020). Therapeutic Targeting of FLT3 and Associated Drug Resistance in Acute Myeloid Leukemia. J. Hematol. Oncol..

[114] Zarnegar-Lumley S., Alonzo T.A., Gerbing R.B., Othus M., Sun Z., Ries R.E., Wang J., Leonti A., Kutny M.A., Ostronoff F. (2023). Characteristics and Prognostic Impact of IDH Mutations in AML: A COG, SWOG, and ECOG Analysis. Blood Adv..

[115] Yang H., Ye D., Guan K.-L., Xiong Y. (2012). IDH1 and IDH2 Mutations in Tumorigenesis: Mechanistic Insights and Clinical Perspectives. Clin. Cancer Res..

[116] Stein E.M., DiNardo C.D., Pollyea D.A., Fathi A.T., Roboz G.J., Altman J.K., Stone R.M., DeAngelo D.J., Levine R.L., Flinn I.W. (2017). Enasidenib in Mutant IDH2 Relapsed or Refractory Acute Myeloid Leukemia. Blood.

[117] DiNardo C.D., Stein E.M., Pigneux A., Altman J.K., Collins R., Erba H.P., Watts J.M., Uy G.L., Winkler T., Wang H. (2021). Outcomes of Patients with IDH1-Mutant Relapsed or Refractory Acute Myeloid Leukemia Receiving Ivosidenib Who Proceeded to Hematopoietic Stem Cell Transplant. Leukemia.

[118] Harding J.J., Lowery M.A., Shih A.H., Schvartzman J.M., Hou S., Famulare C., Patel M., Roshal M., Do R.K., Zehir A. (2018). Isoform Switching as a Mechanism of Acquired Resistance to Mutant Isocitrate Dehydrogenase Inhibition. Cancer Discov..

[119] Intlekofer A.M., Shih A.H., Wang B., Nazir A., Rustenburg A.S., Albanese S.K., Patel M., Famulare C., Correa F.M., Takemoto N. (2018). Acquired Resistance to IDH Inhibition through Trans or Cis Dimer-Interface Mutations. Nature.

[120] DiNardo C.D., Stein E.M., De Botton S., Roboz G.J., Altman J.K., Mims A.S., Swords R., Collins R.H., Mannis G.N., Pollyea D.A. (2018). Durable Remissions with Ivosidenib in IDH1-Mutated Relapsed or Refractory AML. N. Engl. J. Med..

[121] Wang F., Morita K., DiNardo C.D., Furudate K., Tanaka T., Yan Y., Patel K.P., MacBeth K.J., Wu B., Liu G. (2021). Leukemia Stemness and Co-Occurring Mutations Drive Resistance to IDH Inhibitors in Acute Myeloid Leukemia. Nat. Commun..

[122] Amatangelo M.D., Quek L., Shih A., Stein E.M., Roshal M., David M.D., Marteyn B., Farnoud N.R., De Botton S., Bernard O.A. (2017). Enasidenib Induces Acute Myeloid Leukemia Cell Differentiation to Promote Clinical Response. Blood.

[123] Stuani L., Sabatier M., Saland E., Cognet G., Poupin N., Bosc C., Castelli F.A., Gales L., Turtoi E., Montersino C. (2021). Mitochondrial Metabolism Supports Resistance to IDH Mutant Inhibitors in Acute Myeloid Leukemia. J. Exp. Med..

[124] Wong S.H.K., Goode D.L., Iwasaki M., Wei M.C., Kuo H.-P., Zhu L., Schneidawind D., Duque-Afonso J., Weng Z., Cleary M.L. (2015). The H3K4-Methyl Epigenome Regulates Leukemia Stem Cell Oncogenic Potential. Cancer Cell.

[125] Meyer C., Schneider B., Jakob S., Strehl S., Attarbaschi A., Schnittger S., Schoch C., Jansen M.W.J.C., van Dongen J.J.M., den Boer M.L. (2006). The MLL Recombinome of Acute Leukemias. Leukemia.

[126] Issa G.C., Aldoss I., DiPersio J., Cuglievan B., Stone R., Arellano M., Thirman M.J., Patel M.R., Dickens D.S., Shenoy S. (2023). The Menin Inhibitor Revumenib in KMT2A-Rearranged or NPM1-Mutant Leukaemia. Nature.

[127] Perner F., Stein E.M., Wenge D.V., Singh S., Kim J., Apazidis A., Rahnamoun H., Anand D., Marinaccio C., Hatton C. (2023). MEN1 Mutations Mediate Clinical Resistance to Menin Inhibition. Nature.

[128] Adriaanse F.R., Schneider P., Arentsen-Peters S.T., Fonseca A.M.N.D., Stutterheim J., Pieters R., Zwaan C.M., Stam R.W. (2024). Distinct Responses to Menin Inhibition and Synergy with DOT1L Inhibition in KMT2A-Rearranged Acute Lymphoblastic and Myeloid Leukemia. Int. J. Mol. Sci..

[129] Soto-Feliciano Y.M., Sánchez-Rivera F.J., Perner F., Barrows D.W., Kastenhuber E.R., Ho Y.-J., Carroll T., Xiong Y., Anand D., Soshnev A.A. (2023). A Molecular Switch between Mammalian MLL Complexes Dictates Response to Menin-MLL Inhibition. Cancer Discov..

[130] Issa G.C., Aldoss I., Thirman M.J., DiPersio J., Arellano M., Blachly J.S., Mannis G.N., Perl A., Dickens D.S., McMahon C.M. (2025). Menin Inhibition with Revumenib for KMT2A-Rearranged Relapsed or Refractory Acute Leukemia (AUGMENT-101). J. Clin. Oncol..

[131] Heikamp E.B., Henrich J.A., Perner F., Wong E.M., Hatton C., Wen Y., Barwe S.P., Gopalakrishnapillai A., Xu H., Uckelmann H.J. (2022). The Menin-MLL1 Interaction Is a Molecular Dependency in NUP98-Rearranged AML. Blood.

[132] Rasouli M., Blair H., Troester S., Szoltysek K., Cameron R., Ashtiani M., Krippner-Heidenreich A., Grebien F., McGeehan G., Zwaan C.M. (2023). The MLL-Menin Interaction Is a Therapeutic Vulnerability in NUP98-Rearranged AML. Hemasphere.

[133] Janssens D.H., Duran M., Otto D.J., Wu W., Xu Y., Kirkey D., Mullighan C.G., Yi J.S., Meshinchi S., Sarthy J.F. (2024). MLL Oncoprotein Levels Influence Leukemia Lineage Identities. Nat. Commun..

[134] Fukushima N., Minami Y., Kakiuchi S., Kuwatsuka Y., Hayakawa F., Jamieson C., Kiyoi H., Naoe T. (2016). Small-molecule Hedgehog Inhibitor Attenuates the Leukemia-initiation Potential of Acute Myeloid Leukemia Cells. Cancer Sci..

[135] Litingtung Y., Dahn R.D., Li Y., Fallon J.F., Chiang C. (2002). Shh and Gli3 Are Dispensable for Limb Skeleton Formation but Regulate Digit Number and Identity. Nature.

[136] te Welscher P., Zuniga A., Kuijper S., Drenth T., Goedemans H.J., Meijlink F., Zeller R. (2002). Progression of Vertebrate Limb Development through SHH-Mediated Counteraction of GLI3. Science.

[137] Chaudhry P., Singh M., Triche T.J., Guzman M., Merchant A.A. (2017). GLI3 Repressor Determines Hedgehog Pathway Activation and Is Required for Response to SMO Antagonist Glasdegib in AML. Blood.

[138] Pezzotta A., Gentile I., Genovese D., Totaro M.G., Battaglia C., Leung A.Y.-H., Fumagalli M., Parma M., Cazzaniga G., Fazio G. (2022). HDAC6 Inhibition Decreases Leukemic Stem Cell Expansion Driven by Hedgehog Hyperactivation by Restoring Primary Ciliogenesis. Pharmacol. Res..

[139] Delbridge A.R.D., Grabow S., Strasser A., Vaux D.L. (2016). Thirty Years of BCL-2: Translating Cell Death Discoveries into Novel Cancer Therapies. Nat. Rev. Cancer.

[140] Khoo K.H., Verma C.S., Lane D.P. (2014). Drugging the P53 Pathway: Understanding the Route to Clinical Efficacy. Nat. Rev. Drug Discov..

[141] Youle R.J., Strasser A. (2008). The BCL-2 Protein Family: Opposing Activities That Mediate Cell Death. Nat. Rev. Mol. Cell Biol..

[142] Carter B.Z., Tao W., Mak P.Y., Ostermann L.B., Mak D., McGeehan G., Ordentlich P., Andreeff M. (2021). Menin Inhibition Decreases Bcl-2 and Synergizes with Venetoclax in NPM1/FLT3-Mutated AML. Blood.

[143] Zhang Q., Riley-Gillis B., Han L., Jia Y., Lodi A., Zhang H., Ganesan S., Pan R., Konoplev S.N., Sweeney S.R. (2022). Activation of RAS/MAPK Pathway Confers MCL-1 Mediated Acquired Resistance to BCL-2 Inhibitor Venetoclax in Acute Myeloid Leukemia. Signal Transduct. Target. Ther..

[144] Pan R., Ruvolo V., Mu H., Leverson J.D., Nichols G., Reed J.C., Konopleva M., Andreeff M. (2017). Synthetic Lethality of Combined Bcl-2 Inhibition and P53 Activation in AML: Mechanisms and Superior Antileukemic Efficacy. Cancer Cell.

[145] Bhatt S., Pioso M.S., Olesinski E.A., Yilma B., Ryan J.A., Mashaka T., Leutz B., Adamia S., Zhu H., Kuang Y. (2020). Reduced Mitochondrial Apoptotic Priming Drives Resistance to BH3 Mimetics in Acute Myeloid Leukemia. Cancer Cell.

[146] Nechiporuk T., Kurtz S.E., Nikolova O., Liu T., Jones C.L., D’Alessandro A., Culp-Hill R., d’Almeida A., Joshi S.K., Rosenberg M. (2019). The TP53 Apoptotic Network Is a Primary Mediator of Resistance to BCL2 Inhibition in AML Cells. Cancer Discov..

[147] Jones C.L., Stevens B.M., Pollyea D.A., Culp-Hill R., Reisz J.A., Nemkov T., Gehrke S., Gamboni F., Krug A., Winters A. (2020). Nicotinamide Metabolism Mediates Resistance to Venetoclax in Relapsed Acute Myeloid Leukemia Stem Cells. Cell Stem Cell.

[148] Chen X., Glytsou C., Zhou H., Narang S., Reyna D.E., Lopez A., Sakellaropoulos T., Gong Y., Kloetgen A., Yap Y.S. (2019). Targeting Mitochondrial Structure Sensitizes Acute Myeloid Leukemia to Venetoclax Treatment. Cancer Discov..

[149] Zhang H., Nakauchi Y., Köhnke T., Stafford M., Bottomly D., Thomas R., Wilmot B., McWeeney S.K., Majeti R., Tyner J.W. (2020). Integrated Analysis of Patient Samples Identifies Biomarkers for Venetoclax Efficacy and Combination Strategies in Acute Myeloid Leukemia. Nat. Cancer.

[150] Wang E., Pineda J.M.B., Kim W.J., Chen S., Bourcier J., Stahl M., Hogg S.J., Bewersdorf J.P., Han C., Singer M.E. (2023). Modulation of RNA Splicing Enhances Response to BCL2 Inhibition in Leukemia. Cancer Cell.

[151] Han L., Zhang Q., Shi C., Cavazos A., Ruvolo V.R., Leverson J.D., Dail M., Phillips D.C., Chen J., Jin S.S. (2017). Targeting MAPK Signaling Pathway with Cobimetinib (GDC-0973) Enhances Anti-Leukemia Efficacy of Venetoclax (ABT-199/GDC-0199) in Acute Myeloid Leukemia Models. Clin. Lymphoma Myeloma Leuk..

[152] Bestor T.H. (2000). The DNA Methyltransferases of Mammals. Hum. Mol. Genet..

[153] Zhao A., Zhou H., Yang J., Li M., Niu T. (2023). Epigenetic Regulation in Hematopoiesis and Its Implications in the Targeted Therapy of Hematologic Malignancies. Signal Transduct. Target. Ther..

[154] Tsai H.-C., Li H., Van Neste L., Cai Y., Robert C., Rassool F.V., Shin J.J., Harbom K.M., Beaty R., Pappou E. (2012). Transient Low Doses of DNA-Demethylating Agents Exert Durable Antitumor Effects on Hematological and Epithelial Tumor Cells. Cancer Cell.

[155] Messingerova L., Imrichova D., Kavcova H., Turakova K., Breier A., Sulova Z. (2015). Acute Myeloid Leukemia Cells MOLM-13 and SKM-1 Established for Resistance by Azacytidine Are Crossresistant to P-Glycoprotein Substrates. Toxicol. In Vitro.

[156] Valencia A., Masala E., Rossi A., Martino A., Sanna A., Buchi F., Canzian F., Cilloni D., Gaidano V., Voso M.T. (2014). Expression of Nucleoside-Metabolizing Enzymes in Myelodysplastic Syndromes and Modulation of Response to Azacitidine. Leukemia.

[157] Qin T., Jelinek J., Si J., Shu J., Issa J.P.J. (2009). Mechanisms of Resistance to 5-Aza-2′-Deoxycytidine in Human Cancer Cell Lines. Blood.

[158] Gu X., Tohme R., Tomlinson B., Sakre N., Hasipek M., Durkin L., Schuerger C., Grabowski D., Zidan A.M., Radivoyevitch T. (2021). Decitabine- and 5-Azacytidine Resistance Emerges from Adaptive Responses of the Pyrimidine Metabolism Network. Leukemia.

[159] Mahfouz R.Z., Jankowska A., Ebrahem Q., Gu X., Visconte V., Tabarroki A., Terse P., Covey J., Chan K., Ling Y. (2013). Increased CDA Expression/Activity in Males Contributes to Decreased Cytidine Analog Half-Life and Likely Contributes to Worse Outcomes with 5-Azacytidine or Decitabine Therapy. Clin. Cancer Res..

[160] Oellerich T., Schneider C., Thomas D., Knecht K.M., Buzovetsky O., Kaderali L., Schliemann C., Bohnenberger H., Angenendt L., Hartmann W. (2019). Selective Inactivation of Hypomethylating Agents by SAMHD1 Provides a Rationale for Therapeutic Stratification in AML. Nat. Commun..

[161] Penter L., Liu Y., Wolff J.O., Yang L., Taing L., Jhaveri A., Southard J., Patel M., Cullen N.M., Pfaff K.L. (2023). Mechanisms of Response and Resistance to Combined Decitabine and Ipilimumab for Advanced Myeloid Disease. Blood.

[162] Dinndorf P.A., Andrews R.G., Benjamin D., Ridgway D., Wolff L., Bernstein I.D. (1986). Expression of Normal Myeloid-Associated Antigens by Acute Leukemia Cells. Blood.

[163] Pagano L., Fianchi L., Caira M., Rutella S., Leone G. (2007). The Role of Gemtuzumab Ozogamicin in the Treatment of Acute Myeloid Leukemia Patients. Oncogene.

[164] Matsui H., Takeshita A., Naito K., Shinjo K., Shigeno K., Maekawa M., Yamakawa Y., Tanimoto M., Kobayashi M., Ohnishi K. (2002). Reduced Effect of Gemtuzumab Ozogamicin (CMA-676) on P-Glycoprotein and/or CD34-Positive Leukemia Cells and Its Restoration by Multidrug Resistance Modifiers. Leukemia.

[165] Walter R.B., Raden B.W., Hong T.C., Flowers D.A., Bernstein I.D., Linenberger M.L. (2003). Multidrug Resistance Protein Attenuates Gemtuzumab Ozogamicin-Induced Cytotoxicity in Acute Myeloid Leukemia Cells. Blood.

[166] Walter R.B., Raden B.W., Cronk M.R., Bernstein I.D., Appelbaum F.R., Banker D.E. (2004). The Peripheral Benzodiazepine Receptor Ligand PK11195 Overcomes Different Resistance Mechanisms to Sensitize AML Cells to Gemtuzumab Ozogamicin. Blood.

[167] Lamba J.K., Chauhan L., Shin M., Loken M.R., Pollard J.A., Wang Y.C., Ries R.E., Aplenc R., Hirsch B.A., Raimondi S.C. (2017). CD33 Splicing Polymorphism Determines Gemtuzumab Ozogamicin Response in De Novo Acute Myeloid Leukemia: Report from Randomized Phase III Children’s Oncology Group Trial AAML0531. J. Clin. Oncol..

[168] Vago L., Gojo I. (2020). Immune Escape and Immunotherapy of Acute Myeloid Leukemia. J. Clin. Investig..

[169] Davids M.S., Kim H.T., Bachireddy P., Costello C., Liguori R., Savell A., Lukez A.P., Avigan D., Chen Y.B., McSweeney P. (2016). Ipilimumab for Patients with Relapse after Allogeneic Transplantation. N. Engl. J. Med..

[170] Daver N., Alotaibi A.S., Bücklein V., Subklewe M. (2021). T-Cell-Based Immunotherapy of Acute Myeloid Leukemia: Current Concepts and Future Developments. Leukemia.

[171] Rosenblatt J., Stone R.M., Uhl L., Neuberg D., Joyce R., Levine J.D., Arnason J., McMasters M., Luptakova K., Jain S. (2016). Individualized Vaccination of AML Patients in Remission Is Associated with Induction of Antileukemia Immunity and Prolonged Remissions. Sci. Transl. Med..

[172] O’Donnell J.S., Teng M.W., Smyth M.J. (2019). Cancer Immunoediting and Resistance to T Cell-Based Immunotherapy. Nat. Rev. Clin. Oncol..

[173] Wang H., Zhang Q., Teng Q., Li Z., Liu H., Wang Z.M., Li B., Xia Y., Jin J. (2023). A Phase 1b Study Evaluating the Safety and Efficacy of AK117 (Anti-CD47 Monoclonal Antibody) in Combination with Azacitidine in Patients with Treatment-Naïve Acute Myeloid Leukemia. Blood.

[174] Mesaros O., Onciul M., Matei E., Joldes C., Jimbu L., Neaga A., Serban O., Zdrenghea M., Nanut A.M. (2024). Macrophages as Potential Therapeutic Targets in Acute Myeloid Leukemia. Biomedicines.

[175] Greiner J., Götz M., Hofmann S., Schrezenmeier H., Wiesneth M., Bullinger L., Döhner H., Schneider V. (2020). Specific T-Cell Immune Responses against Colony-Forming Cells Including Leukemic Progenitor Cells of AML Patients Were Increased by Immune Checkpoint Inhibition. Cancer Immunol. Immunother..

[176] Lichtenegger F.S., Krupka C., Haubner S., Köhnke T., Subklewe M. (2017). Recent Developments in Immunotherapy of Acute Myeloid Leukemia. J. Hematol. Oncol..

[177] Cunningham I., Gee T., Reich L., Kempin S., Naval A., Clarkson B. (1989). Acute Promyelocytic Leukemia: Treatment Results during a Decade at Memorial Hospital. Blood.

[178] Yilmaz M., Kantarjian H., Ravandi F. (2021). Acute Promyelocytic Leukemia Current Treatment Algorithms. Blood Cancer J..

[179] Vadakekolathu J., Minden M.D., Hood T., Church S.E., Reeder S., Altmann H., Sullivan A.H., Viboch E.J., Patel T., Ibrahimova N. (2020). Immune Landscapes Predict Chemotherapy Resistance and Immunotherapy Response in Acute Myeloid Leukemia. Sci. Transl. Med..

[180] Jin J., Hou S., Yao Y., Liu M., Mao L., Yang M., Tong H., Zeng T., Huang J., Zhu Y. (2024). Phosphoproteomic Characterization and Kinase Signature Predict Response to Venetoclax Plus 3+7 Chemotherapy in Acute Myeloid Leukemia. Adv. Sci..

[181] Moncada R., Barkley D., Wagner F., Chiodin M., Devlin J.C., Baron M., Hajdu C.H., Simeone D.M., Yanai I. (2020). Integrating Microarray-Based Spatial Transcriptomics and Single-Cell RNA-Seq Reveals Tissue Architecture in Pancreatic Ductal Adenocarcinomas. Nat. Biotechnol..

[182] Glytsou C., Chen X., Zacharioudakis E., Al-Santli W., Zhou H., Nadorp B., Lee S., Lasry A., Sun Z., Papaioannou D. (2023). Mitophagy Promotes Resistance to BH3 Mimetics in Acute Myeloid Leukemia. Cancer Discov..

[183] Burd A., Levine R.L., Ruppert A.S., Mims A.S., Borate U., Stein E.M., Patel P., Baer M.R., Stock W., Deininger M. (2020). Precision Medicine Treatment in Acute Myeloid Leukemia Using Prospective Genomic Profiling: Feasibility and Preliminary Efficacy of the Beat AML Master Trial. Nat. Med..

[184] Yamaura T., Nakatani T., Uda K., Ogura H., Shin W., Kurokawa N., Saito K., Fujikawa N., Date T., Takasaki M. (2018). A Novel Irreversible FLT3 Inhibitor, FF-10101, Shows Excellent Efficacy against AML Cells with FLT3 Mutations. Blood.

[185] Mellinghoff I.K., van den Bent M.J., Blumenthal D.T., Touat M., Peters K.B., Clarke J., Mendez J., Yust-Katz S., Welsh L., Mason W.P. (2023). Vorasidenib in IDH1- or IDH2-Mutant Low-Grade Glioma. N. Engl. J. Med..

[186] Desikan S.P., Daver N., DiNardo C., Kadia T., Konopleva M., Ravandi F. (2022). Resistance to Targeted Therapies: Delving into FLT3 and IDH. Blood Cancer J..

[187] Yeung Y.A., Krishnamoorthy V., Dettling D., Sommer C., Poulsen K., Ni I., Pham A., Chen W., Liao-Chan S., Lindquist K. (2020). An Optimized Full-Length FLT3/CD3 Bispecific Antibody Demonstrates Potent Anti-Leukemia Activity and Reversible Hematological Toxicity. Mol. Ther..

[188] Loghavi S., Wei Q., Ravandi F., Quesada A.E., Routbort M.J., Hu S., Toruner G.A., Wang S.A., Wang W., Miranda R.N. (2024). Optical Genome Mapping Improves the Accuracy of Classification, Risk Stratification, and Personalized Treatment Strategies for Patients with Acute Myeloid Leukemia. Am. J. Hematol..

